# Lessons Learned From the Clinical Presentation of Common Variable Immunodeficiency Disorders: A Systematic Review and Meta-Analysis

**DOI:** 10.3389/fimmu.2021.620709

**Published:** 2021-03-23

**Authors:** Lisanne M. A. Janssen, Michiel van der Flier, Esther de Vries

**Affiliations:** ^1^ Department of Tranzo, Tilburg University, Tilburg, Netherlands; ^2^ Department of Pediatrics, Amalia Children’s Hospital, Nijmegen, Netherlands; ^3^ Department of Pediatric Infectious Diseases and Immunology, Wilhelmina Children’s Hospital, University Medical Center Utrecht, Utrecht, Netherlands; ^4^ Laboratory of Medical Microbiology and Immunology, Elisabeth-Tweesteden Hospital, Tilburg, Netherlands

**Keywords:** humoral immunodeficiency, antibody deficiency, common variable immunodeficiency disorders, non-infectious complications, clinical manifestations

## Abstract

**Background:**

Diagnostic delay in common variable immunodeficiency disorders (CVID) is considerable. There is no generally accepted symptom-recognition framework for its early detection.

**Objective:**

To systematically review all existing data on the clinical presentation of CVID.

**Methods:**

PubMed, EMBASE and Cochrane were searched for cohort studies, published January/1999-December/2019, detailing the clinical manifestations before, at and after the CVID-diagnosis.

**Results:**

In 51 studies (n=8521 patients) 134 presenting and 270 total clinical manifestations were identified. Recurrent upper and/or lower respiratory infections were present at diagnosis in 75%. Many patients had suffered severe bacterial infections (osteomyelitis 4%, meningitis 6%, septicemia 8%, mastoiditis 8%). Bronchiectasis (28%), lymphadenopathy (27%), splenomegaly (13%), inflammatory bowel disease (11%), autoimmune cytopenia (10%) and idiopathic thrombocytopenia (6%) were also frequently reported. A bimodal sex distribution was found, with male predominance in children (62%) and female predominance in adults (58%). 25% of CVID-patients developed other manifestations besides infections in childhood, this percentage was much higher in adults (62%). Immune-dysregulation features, such as granulomatous-lymphocytic interstitial lung disease and inflammatory bowel disease, were more prominent in adults.

**Conclusions:**

The shift from male predominance in childhood to female predominance in adults suggests differences in genetic and environmental etiology in CVID and has consequences for pathophysiologic studies. We confirm the high frequency of respiratory infections at presentation, but also show a high incidence of severe bacterial infections such as sepsis and meningitis, and immune dysregulation features including lymphoproliferative, gastrointestinal and autoimmune manifestations. Early detection of CVID may be improved by screening for antibody deficiency in patients with these manifestations.

## Introduction

Common variable immunodeficiency disorders (CVID) is a collection of heterogeneous clinical manifestations linked by low serum levels of immunoglobulins and primary failure of specific antibody production (1–3). The rates of serious comorbidities and resulting mortality of patients with CVID drastically exceed the respective rates in the general population, imposing a high disease burden to the individual patient (4, 5). Although CVID is the most common symptomatic primary immunodeficiency (PID), it is still a rare disease with a greatly varying observed prevalence between countries, ranging in “industrialized countries” from 6.9/100,000 in Finland to 0.6/100,000 in Spain (6–14) and even lower observed prevalence rates (<0.5/100,000) in “developing” countries (15). Therefore, CVID has a low prevalence in primary care and general hospital settings, where non-immunologists have little knowledge of this disease. Also, respiratory infections and non-infectious complications of CVID such as lymphoproliferation, granulomatous disease and autoimmunity are much more prevalent *without* concomitant CVID. This makes it challenging to front-line clinicians to recognize CVID in these cases. Because of the variability of presenting clinical manifestations, patients visit various physicians of different specialties in search of a diagnosis, which increases the risk of missing the overarching clinical pattern and thereby overlooking the underlying hypogammaglobulinemia (16).

Timely diagnosis and optimal management are likely to result in improved clinical and quality-of-life outcomes for patients with CVID, higher participation in society (school, work) and lower health care costs (4, 17–19). Reducing diagnostic delay is therefore crucial; current approaches mainly comprise improving education and awareness of clinicians in both primary and secondary care. Already a long time ago, the Jeffrey Modell Foundation (JMF) developed ten (mainly pediatric) (20) and the European Society for Immunodeficiencies (ESID) six (adult) ‘Warnings Signs’ to indicate PIDs (21). Unfortunately, these signs have turned out to have a low sensitivity for timely PID diagnosis (22, 23).

In order to improve our insight in the early presentation of CVID and to assist physicians in its timely detection, we aimed to systematically identify and collate existing published cohort studies on the presenting clinical manifestations at and before diagnosis. In addition, we included the overall clinical manifestations during disease follow-up in our systematic review; this was done separately for children and adults to evaluate age-related differences and similarities in pediatric and adult onset CVID.

## Methods

### Search Strategy

We searched EMBASE, Cochrane and PubMed from January 1999 to December 2019 (inclusive) using a combination of subject headings and free text incorporating the terms ‘common variable immunodeficiency’, ‘late onset hypogammaglobulinemia’, and ‘diagnosis’, and limited to English language and humans. Reference lists of included studies were also searched for potentially relevant studies (snowball method). The complete search strategy is detailed in the supplementary appendix eSearch. The protocol of this systematic review has been registered on PROSPERO with registration number CRD42019121384.

### Study Selection

We considered all primary research studies for selection, either retrospective or prospective, of any study design (e.g., case series, cohort), describing the clinical manifestations for a minimum of 10 patients with CVID. Two researchers (LJ and EV) independently screened titles and abstracts of all papers, excluding clearly irrelevant studies. Hereafter, they independently reviewed the full text of remaining papers to assess eligibility. If multiple updates of a cohort were published, the most recent study with the largest dataset describing the total clinical picture of their CVID cohort was included, in order to avoid duplicates of patients in our review. The large European multicenter study by Gathmann et al. (24) was excluded for analysis to avoid overlapping data, because this study collated data from multiple centers that already published a substantial amount of their data as single-center cohorts in more detail. Three European multicenter studies (25–27) partially overlapped in their included centers; in this case the largest multicenter study by Chapel et al. describing the overall clinical picture of CVID was included (26) (for details about the handling of overlapping data, see **Supplementary Table 1**). Studies that selected cases based on the presence of only certain clinical features of CVID (e.g., only granulomatous, pulmonary, gastrointestinal or autoimmune manifestations) were excluded to avoid giving disproportionate weight to those features in the data synthesis, unless the total number of CVID patients from which these cases were selected was also reported. When the same center/registry published an article about their total cohort and another article in which children and/or adults were separately described, these children- and adult-specific overlapping data were only included in the subgroup analysis for children vs adults. Any uncertainties regarding study selection were discussed between LJ, MF, and EV.

### Quality Assessment

After assembling a shortlist of studies eligible for potential inclusion, LJ assessed the risk of bias in these studies to ensure that only those studies with an acceptable risk of bias were included. This quality assessment was checked by EV. Because there is no validated quality checklist for assessing retrospective cohort studies, we constructed a checklist based on relevant items from the MOOSE (meta-analysis of observational studies in epidemiology) reporting guideline for observational studies (28), the STROBE (strengthening the reporting of observational studies in epidemiology) reporting guideline for cohort studies (29) and CASP (critical appraisal skills program) guidelines for case-control and cohort studies (checklist in **Supplementary Table 2**) (30). Quality was assessed as ‘acceptable’ or ‘unacceptable’ in three domains: definition of CVID, selection of cases, and methods for extracting data on included cases. ‘Acceptable’ for case definition required cases to be defined according to the diagnostic criteria of ESID/Pan-American Group for Immunodeficiency (PAGID) (3), the ESID Registry working definitions for clinical diagnosis of PID (www.esid.org), the International Union of Immunological Societies (IUIS) criteria (31), the World Health Organization (WHO) scientific group (32) or the international consensus document (ICON) (33) (**Supplementary Table 3**), or - if no reference was made to which diagnostic criteria were used - description of the inclusion criteria corresponding to the above described diagnostic criteria. Although we only included articles about pediatric CVID that reported to have only included established CVID patients, we cannot completely rule out that a few of these patients actually had transient hypogammaglobulinemia of infancy. ‘Acceptable’ for case selection required that at least two of the participants’ baseline characteristics were clearly documented and that the characteristics of cases were sufficiently consistent with the current knowledge regarding CVID (i.e., the age and sex distribution of cases matched the known epidemiology of CVID). ‘Acceptable’ for data extraction required the use of a standardized data collection format and/or the objective measurement of signs (e.g., CT confirmation of bronchiectasis, biopsy confirmation of granulomas). Disagreements between the two reviewers (LJ and EV) were discussed with a third reviewer (MF) until agreement was achieved. Only studies considered by the two reviewers to be acceptable for case definition and to pass in at least one other domain were included, and data related to the ‘unacceptable’ domain were not included in this review.

### Data Extraction

Data were extracted from included studies by LJ using a standardized Microsoft Excel spread sheet and were checked by EV and MF. We extracted study characteristics including year of publication, country, recruitment periods, number and type of centers, study design, number of patients, age and sex. Clinical manifestations were recorded, and numbers of patients with each manifestation were noted. This was done separately for the clinical manifestations at presentation (at or before diagnosis) and overall (at any timepoint). When this distinction was not described, the manifestations were collected as ‘overall’ in the standardized format. When a clinical manifestation was not discussed in a study, we made no assumption about whether or not that manifestation had occurred in that population but recorded this item as ‘missing data’ for the respective study in the standardized format. We deliberately chose to use the exact wordings of the included studies, to avoid interpretation bias. Clinical manifestations were never counted twice. For example, where one study separately described sinusitis and otitis, another study only mentioned ‘upper respiratory tract infections’. In addition, we recorded whether in a cohort, children, adults or both were described. A pediatric cohort was defined as age during follow-up <18 years old, which was comparable to the cut-off value for children’s age provided by the original studies. We did not contact the authors of included papers to collect additional information.

### Statistical Analysis

We used MetaXL (version 5.3, EpiGear International, Queensland, Australia) to calculate proportions and standard errors (SEs) of proportions for each clinical manifestation in each included study (34). To combine the results of multiple cohorts, we calculated pooled proportions of each clinical manifestation using the metan command. Anticipating high heterogeneity between included studies, we performed random effects meta-analysis using the DerSimonian and Laird method and standard methods to calculate I^2^ as an estimate of heterogeneity. In addition, we conducted two subgroup analyses using the same techniques: 1) children vs adults, and 2) clinical manifestations at presentation vs overall clinical manifestations during the disease course. A subgroup analysis based on age was conducted because the few available studies on differences between pediatric-onset and adult-onset CVID have yielded limited and conflicting data (35–37). In order to improve our understanding of the early presentation of CVID, which can assist clinicians in timely detection of this condition, we focused our analysis on clinical manifestations at or prior to diagnosis.

## Results

### Search Results

**Figure 1:**
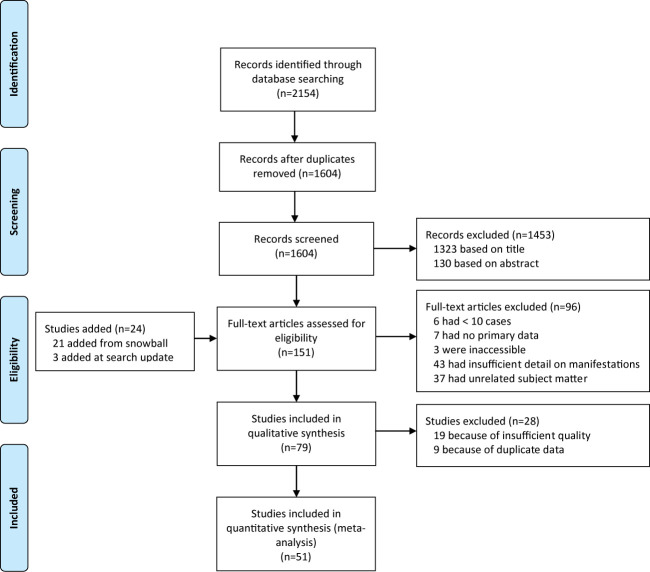
Flow chart showing the study selection process.

After removal of duplicates, we identified 1604 papers. We excluded 1453 after screening titles and abstracts, and a further 96 after full-text assessment (**Figure 1**), based on the inclusion criteria (see Method). Reference lists of included studies yielded 21 additional eligible studies. There was full consensus between the authors regarding study inclusion.

### Characteristics of Included Studies

The 51 included studies described clinical manifestations in a total of 8521 patients (**Table 1**) (5, 6, 19, 26, 35–81). 50 studies were conducted in one or more centers in one country only, in 18 different countries in total; 1 study included multiple centers from different countries (26). Most were cohort studies (5 prospective, 42 retrospective) and three compared cases with controls. All 51 studies extracted data from written/typed hospital records. The majority of studies (n=39) identified cases from hospital records alone; others also used regional, national, or continental registries of primary immunodeficiencies (n=12). Three studies also obtained data from a patient and/or parent-completed questionnaire (41, 45, 55). Fifteen studies reported clinical manifestations of their total CVID cohort, but reported in more detail on patients based on the presence of only certain clinical features of CVID: asthma and allergic diseases (39, 81), autoimmune manifestations (63, 65, 66), gastric cancer (49), gastrointestinal manifestations (41, 76), granulomatous manifestations (64, 78), or pulmonary manifestations (43, 45, 46, 69, 75).

**Table 1:** Characteristics of included studies.

Ref	Country			Recruitment period	Source of data	Nr of centers	Nr of pt	Age at diagnosis (years)			Diagnostic delay (years)			Follow-up (years)			Mortality
								Median	Mean	Range	Median	Mean	Range	Median	Mean	Total	
*Adults*																	
(38)		USA		1998-2013	Hospital records	1	34	–	–	–	–	–	–	–	–	–	–
(39)		USA		2008-2018	Hospital records	1	153	–	–	–	–	–	–	–	–	–	–
(40)		USA		1988-2016 2006-2017	USIDNET registry^*^ Partners^$^ research patient data registry	- 3	571 205	- 42	- -	- -	- -	- -	- -	- -	- -	- -	4.1%^a^ 15.1%
(37)		Argentina		1997-2008	Hospital records	1	10	41	–	18-69	–	9.5	–	–	–	–	–
(41)		Norway		–	1)Hospital records 2)”GI symptoms” questionnaire	1	104	–	–	–	–	–	–	–	–	–	–
(42)		Russia		1990-2011	Hospital records	1	57	–	–	–	–	–	–	–	–	5	21%
(43)		The Netherlands		2008-2012	Hospital records	1	47	27	–	–	9.5	–	–	9.5	–	–	–
(44)		France		2004-2007	French DEFI database^*^	31	252	33.9	–	–	6.9	–	0-55	–	–	–	–
(5)		USA		1986-2011	Hospital records	1	473	–	–	–	–	–	–	–	–	40	19.6%
(35)		USA		1988-2016	USIDNET registry^*^	–	264	–	–	18-76.9	–	–	–	–	–	–	–
(45)		United Kingdom		2014-2015	1)Daily checkbox symptom diaries 2)St George’s Respiratory Questionnaire 3)Hospital records	1	134	–	–	–	–	–	–	–	–	–	–
(46)		USA		1985-2001	Hospital records	1	69	–	–	–	–	–	–	–	–	–	–
(47)		Brazil		1980-2003	Hospital records	1	71	–	30.9	–	–	10.9	–	4.5	–	28.3	15.5%
(48)		France		–	Hospital records	1	57	–	–	–	–	–	–	–	–	–	–
(36)		USA		2005-2016	Hospital records	1	107	–	45	–	–	–	–	–	5.7	–	4%
(49)		Italy		2001-2017	Hospital records	3	455	–	40.1	–	–	–	–	–	11.5	–	17.1%
*Children*																	
(50)			Argentina	–	Hospital records	1	28	11.2	11.1	4-16.1	–	5.4	–	–	–	–	–
(51)			Taiwan	1990-2010	Hospital records	1	10	–	4.5	–	–	1.5	–	–	9.8	–	0%
(40)			USA	1988-2016	USIDNET registry^*^	–	212	–	–	–	–	–	–	–	–	–	–
(37)			Argentina	1997-2008	Hospital records	1	21	8.5	–	3-17	–	4.5	–	–	–	–	–
(52)			Spain	1985-2005	Hospital records	1	22	7.8	–	2.5-16	–	–	–	–	–	18	–
(53)			Iran	1984-2010	Hospital records	1	69	–	6.76	4-16	–	4.4	–	–	5.2	21	21.7%
(54)			Poland	1995-2011	Hospital records	1	49	8.8	–	2.4-17.3	2.4	–	0-12.2	–	–	–	–
(35)			USA	1988-2016	USIDNET registry^*^	–	193	–	–	2-17	–	–	–	–	–	–	–
(55)			Germany	1990-2004	1)Hospital records 2)Parent/patient-completed data	1	32	10.4	–	1.1-17.4	5.8	–	0.2-14.3	–	–	–	–
(56)			The Netherlands	1995-2008	Hospital records	1	38	–	5.5	0.9-12.7	–	–	–	–	–	–	–
(57)			USA	–	Hospital records	1	45	–	–	2-16	–	–	–	–	–	–	–
(58)			USA	1992-2005	Hospital records	1	12	8	8.3	2-17	–	–	–	–	–	–	–
(59)			Turkey	2003-2014	Hospital records	1	28	5.9	6.7	1-15	–	–	–	–	–	–	–
(36)			USA	2005-2016	Hospital records	1	91	–	12	–	–	–	–	–	8.6	–	13%
*All ages together*																	
(60)			Iran	1984-2013	Registry database*	14	173	–	12.3	4-54	4	–	0.25-39	–	–	29	30%
(61)			Turkey	2001-2008	Hospital records	1	23	30.5	33	13-73	–	–	1-32	–	–	–	–
(62)			Iran	n/a-2014	Hospital records	1	47	–	20.2	–	–	9	–	–	6.8	23	6%
(63)			France	2004-2008	French DEFI database^*^	–	311	35.2	–	16-58	–	–	–	–	–	–	–
(64)			France	–	French DEFI database^*^	–	436	–	–	–	–	–	–	–	–	–	–
(26)			Multiple	1996-2006	ESID registry^#^	7	334	33	35.3	–	5	7.5	0-61	22.5	25.6	–	14.5%
(65)			USA	n/a-2017	USIDNET registry^*^	50	990	–	–	–	–	–	–	–	–	–	–
(66)			France	2013-2016	CEREDIH registry^*^	–	408	–	–	–	–	–	–	–	–	–	–
(67)			The Netherlands	–	Hospital records	1	32^b^	34.3	–	0-63	–	–	–	15.8	–	–	2.9%
(68)			Finland	1996-1998	1)Central register 2)Hospital records of 5 university hospitals^*^	6	95	33	32	0.5-73	5	8.5	0.2-37	–	–	–	4%
(69)			Spain	–	Hospital records	1	19	–	23.2	–	–	–	–	–	–	–	–
(70)			Iran	1983-2013	Hospital records	1	125	8.3	–	0-54	4	–	0-51	–	–	25	27.2
(71)			Turkey	2008-2014	Hospital records	1	31	23	–	–	14	–	–	–	–	–	–
(72)			Italy	1999-2005	Italian PID Network^*^	26	224	–	26.6	2-73	–	8.9	–	11.5	11.5	34	6%
(73)			Mexico	–	Hospital records^*^	7	43	19	–	–	–	12.5	–	–	–	–	–
(74)			Puerto Rico	–	Hospital records	1	20	–	–	5-30	–	–	–	–	–	–	–
(75)			United Kingdom	1997-1998	Hospital records	1	47	35	–	5-72	4	–	0.8-25	–	–	12	17%
(76)			Iran	1997-2004	Iranian PID registry	1	39	12	16	3-55	–	–	–	–	–	–	–
(77)			USA	1973-1998	Hospital records	1	248	–	31	3-79	–	–	–	7	–	25	27%
(78)			USA	–	Hospital records	2	455	26	–	2-59	–	–	–	–	–	25	20.5
(79)			USA	2011-2015	Hospital records	1	128	–	–	–	–	–	–	–	–	–	–
(19)			Italy	1985-2015	Hospital records	1	75	40	–	–	7	–	–	9	10.24	30	5.3%
(6)			Finland	2007-2015	Hospital records^$^	3	106	–	–	–	–	–	–	–	–	–	9.4%
(80)			Poland	1990-2017	Internet database^$^	4	77	–	32.29	–	–	10,13	–	–	4.26	–	–
(81)			Iran	–	Hospital records	1	187	–	–	–	–	–	–	–	–	–	–

^*^Nationwide. ^#^Continentwide. ^$^Regionwide.

a This is the mortality percentage of the total USIDNET cohort (n = 884).

b Two patients with thymoma were excluded (Good syndrome).

CEREDIH, Centre de Référence Déficits Immunitaires Héréditaires; ESID, European society for immunodeficiency; FU, follow-up; GI, gastro-intestinal; Nr, number; PID, primary immunodeficiency; pt, patients; USIDNET, United States Immunodeficiency Network.

### Risk of Bias of Included Studies

Most included studies defined cases using the diagnostic criteria of PAGID and ESID (27 studies); other used criteria were: the ESID Registry working diagnosis criteria (8 studies), International consensus document (3 studies), IUIS criteria (2 studies), and WHO classification (4 studies). Seven studies did not report which criteria were used but did describe a CVID diagnosis that corresponded to the above approved classifications. One study reported to use both the diagnostic criteria of PAGID/ESID and the WHO classification (51). Lack of routine B and T cell immunophenotyping in most studies prohibited an accurate assessment of potential late-onset combined immunodeficiency (LOCID). 37 studies (73%) included all consecutive cases within the study period, with a further 11 studies (22%) describing why a proportion of potentially eligible cases were excluded. In the remaining 3 studies (6%), the proportion of consecutively included cases was unclear.

A weakness of the included studies was lack of clarity at which point in the diagnostic and follow-up pathway clinical features were recorded. Twenty studies explicitly stated when clinical manifestations occurred [at or before diagnosis (n=4), and both at/or before diagnosis and during follow-up (n=16)]. The remaining 31 studies were unclear as to when the reported clinical manifestations occurred during the disease course.

### Pooled Frequencies of Clinical Characteristics From Meta-Analysis

Pooled frequencies of demographic information are shown in **Table 2**. In pediatric CVID patients, males were in the majority (62%, 95% CI 54-69), while females predominated in the adult CVID patients (58%, 95% CI 53-64). The high pooled proportion of consanguinity in the pediatric and total cohort should be interpreted with caution (31% and 20% respectively; only one study reported this for adults). This proportion varied substantially per country. In an Argentinian cohort none had a history of consanguinity (37), while the rate of consanguinity was very high in an Iranian cohort (72%) (53).

**Table 2:** Demographic parameters in adult-, pediatric-, and total cohorts.

	****Children			****Adults			****Total cohort		
	Number of patients	Pooled proportion (95% CI)	I squared	Number of patients	Pooled proportion (95% CI)	I squared	Number of patients	Pooled proportion (95% CI)	I squared
Males	305/526	62 (54-69)	62	571/1326	43 (37-48)	71	1819/3828	50 (47-54)	71
Females	221/526	38 (31-46)	62	755/1326	58 (53-64)	72	2009/3828	50 (46-53)	73
Family members with PID	31/291	10 (6-15)	41	83/497	14 (9-20)	56	168/1283	12 (9-16)	66
Consanguinity	60/118	31 (0-87)	96	10	0^a^		238/769	20 (4-43)	97

PID, primary immunodeficiency disease.

a Meta-analysis could not be conducted because the feature was described in only one study.

In total, 147 out of a potential of 270 meta-analyses were conducted. For the remaining 123 clinical manifestations, meta-analysis was not possible since the features were each reported in only one study. The high heterogeneity (I^2^) statistics in the meta-analyses (mostly >80%) indicated that the degree of heterogeneity between studies was greater than that expected by chance alone and confirmed the appropriateness of random-effects meta-analysis to generate pooled proportions.

**Figure 2:**
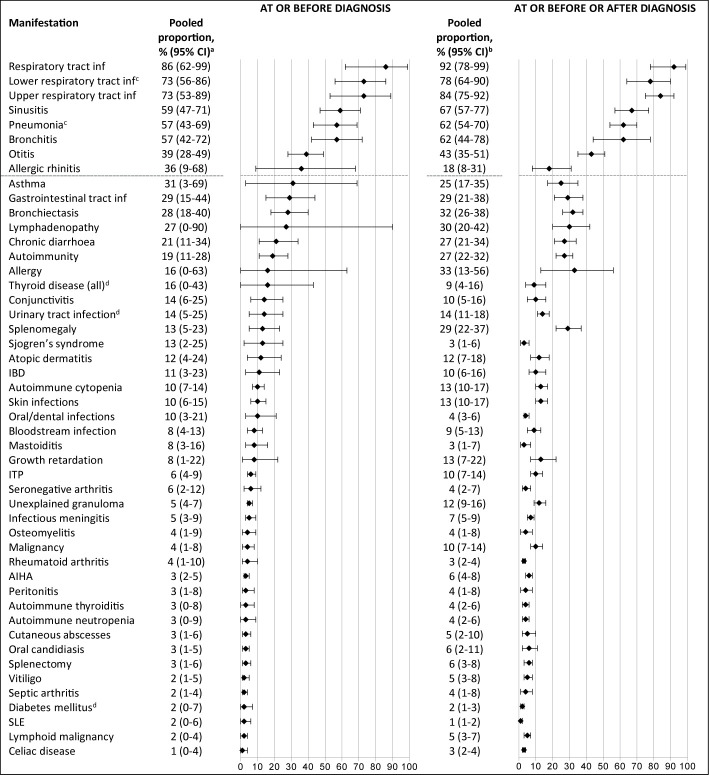
Frequency of reported clinical manifestations at presentation vs overall clinical manifestations during the disease course.The most frequent clinical manifestations at presentation (reported in ≥39% of patients) are shown above the grey dotted horizontal lines. ^a^Number of patients ranged from 44 to 1137; number of studies ranged from 2-15. ^b^Number of patients ranged from 51 to 4061; number of studies ranged from 2-31. ^c^Pneumonia and lower respiratory tract infections were not combined into one category, as they were often mentioned as two separate categories in the included studies. ^d^The prevalence of this clinical manifestation is similar or lower to lifetime prevalence estimates in general population. IBD, inflammatory bowel disease; inf, infections; ITP, idiopathic thrombocytopenic purpura; AIHA, autoimmune hemolytic anemia; SLE, systemic lupus erythematosus.

There were 49 specific clinical manifestations for which it was possible to calculate pooled proportions for the subgroup *at presentation*, i.e. ‘at or before diagnosis’; these are shown in **Figure 2** in comparison with *overall*, i.e. ‘at, before or after’ diagnosis. The most frequent clinical manifestations at presentation (reported in ≥39% of patients) are shown above the grey dotted horizontal lines. A history of upper and/or lower respiratory infections was present at diagnosis in three-quarters of patients (upper respiratory tract infections in 73%, lower respiratory tract infections in 73%, sinusitis in 59%, pneumonia in 57%, bronchitis in 57% and otitis in 39%) and severe bacterial infections in 8% (septicemia), 8% (mastoiditis), 6% (meningitis), and 4% (osteomyelitis).

Bronchiectasis was already present in almost one third of the patients at or before the CVID diagnosis was made (28%, 95% CI 18-40). Non-infectious manifestations that were frequently present at diagnosis were: lymphadenopathy (27%), splenomegaly (13%), inflammatory bowel disease (11%), and autoimmune hematological manifestations (autoimmune cytopenia (10%) and idiopathic thrombocytopenia (6%)). The pooled prevalences at presentation of urinary tract infection (14%, 95% CI 5-25), thyroid disease (16%, 95% CI 0-43), and diabetes mellitus (2%, 95% CI 0-7) correspond to the estimated lifetime prevalence estimates in the general population of 30% (82), 12% (83), and 0.9% (type 1) (84), respectively. An overview of all reported clinical manifestations at and before diagnosis with – when known – lifetime prevalence estimates from the general population is included in **Supplementary Table 4**.

**Figure 3:**
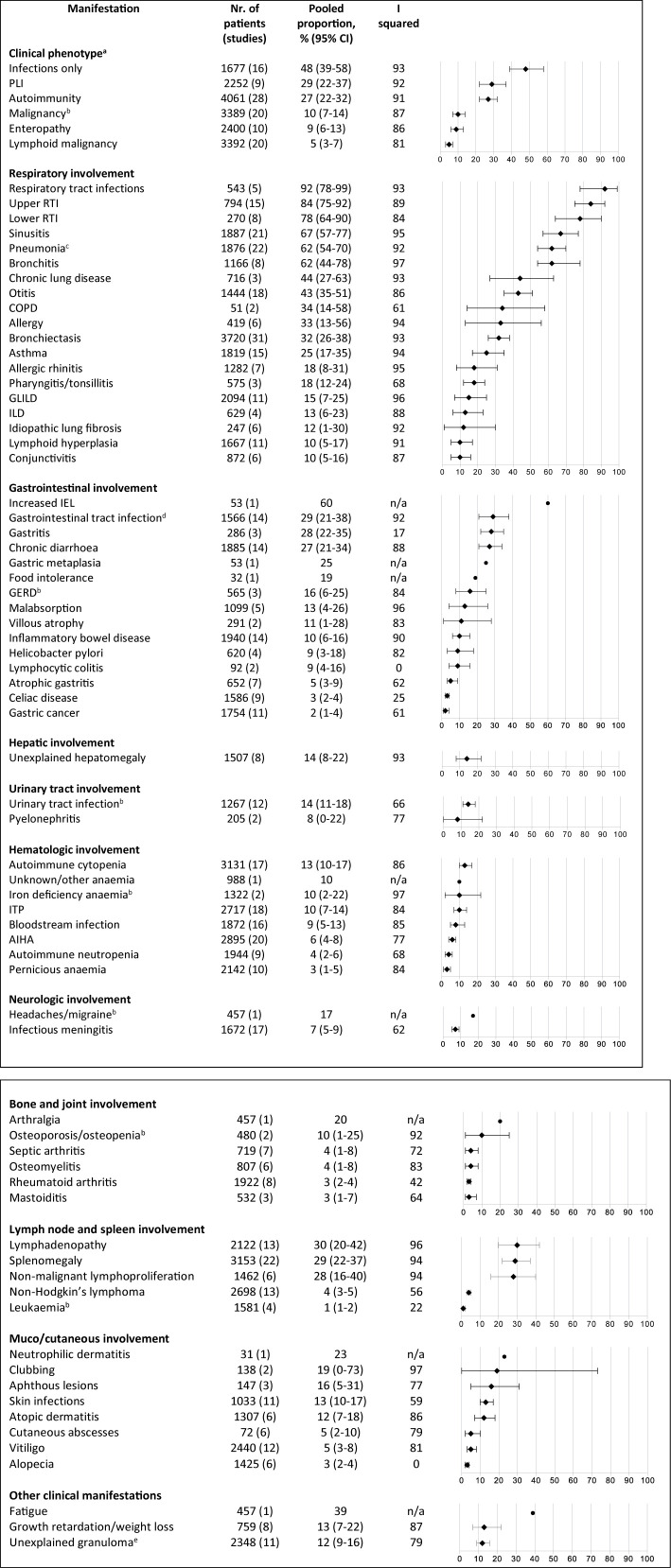
Frequency of reported clinical manifestations of patients with common variable immunodeficiency. All clinical manifestations that were present in ≥10% of patients are shown; the CVID-associated manifestations are also shown when present in <10% when they were considered important to incorporate by the authors (based on current literature and consensus in the field). ^a^Derived from Chapel et al. (26). ^b^The prevalence of this clinical manifestation is similar or lower to lifetime prevalence estimates in general population. ^c^Streptococcus pneumoniae 15%, 95% CI 8-23; Hemophilus influenzae 19%, 95% CI 8-33; Moraxella catarrhalis 7%, 95% CI 0-19; Staphylococcus aureus 7%, 95% CI 3-12; Mycobacterial infection 1%, 95% CI 0-2; Pneumocystis jiroveci 1%, 95% CI 0-2%; Pseudomonas 6%, 95% CI 2-10; Aspergillus 3%, 95% CI 1-5; Mycoplasma 2%, 95% CI 0-4. ^d^Giardia intestinalis 13%, 95% CI 7-21; Candida species 10%, 95% CI 4-19; Salmonella species 6%, 95% CI 2-12; Campylobacter species 4%, 95% CI 1-8.^e^Intestinal granulomatosis 1%, 95% CI 0-4; liver granuloma 3%, 95% CI 1-6; granuloma in lymph node 2%, 95% CI 0-5; granuloma in spleen 1%, 95% CI 0-2; skin granuloma 1%, 95% CI 0-2. AIHA, autoimmune hemolytic anemia; COPD, chronic obstructive pulmonary disease; CVID, common variable immunodeficiency disorders; GERD, gastro-esophageal reflux disease; GLILD, granulomatous and lymphocytic interstitial lung disease; IEL, increased intraepithelial lymphocytes; ILD, interstitial lung disease; ITP, idiopathic thrombocytopenic purpura; PLI, polyclonal lymphocytic infiltration; RTI, respiratory tract infection.

In **Figure 3**, all clinical manifestations that were present in ≥10% of patients are shown (as presented, whether or not likely related to CVID); the CVID-associated manifestations are also shown when present in <10% when they were considered important to incorporate by the authors (based on obvious relation with CVID in the current literature or consensus in the field). We grouped these manifestations into eleven distinct clinical categories according to the body system affected and the clinical phenotypes described by Chapel et al. (26). An overview of all reported clinical manifestations is included in **Supplementary Table 5**. Many CVID patients developed non-infectious manifestations during follow-up: bronchiectasis in 32%, lymphadenopathy in 30%, splenomegaly in 29%, polyclonal lymphocytic infiltration in 29%, and autoimmune manifestations in 27%. In addition, a substantial number of patients developed malignancies (10%) and atopic diseases during the entire disease course [asthma (25%), allergic rhinitis (18%)].

**Figure 4:**
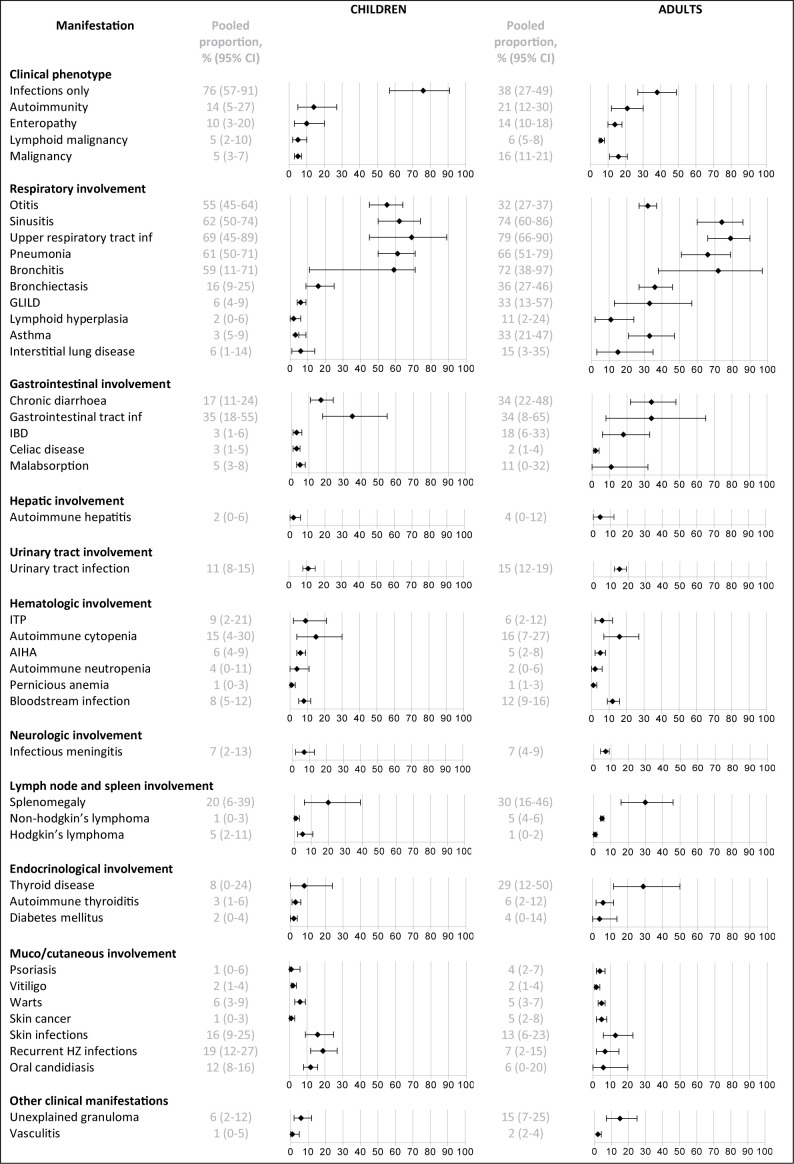
Frequency of reported clinical manifestations in children vs adults. IBD, inflammatory bowel disease; inf, infections; GLILD, granulomatous and lymphocytic interstitial lung disease; ITP, idiopathic thrombocytopenic purpura; AIHA, autoimmune hemolytic anemia.

Three quarters of the children (76%, 95% CI 57-91) developed no other complications besides infections during the reported follow-up periods (See **Figure 4**), while this percentage was much lower in the adults (38%, 95% CI 27-49). Certain infectious features of CVID, such as otitis, were more common in children (55%, 95% CI 45-64) than in adults (32%, 95% CI 27-37), whereas certain immune dysregulation features, such as granulomatous-lymphocytic interstitial lung disease, chronic diarrhea and inflammatory bowel disease were more prominent in adults (33%, 95% CI 13-57; 34%, 95% CI 22-48; 18%, 95% CI 6-33; respectively) than in children (6%, 95% CI 4-9; 17%, 95% CI 11-24; 3%, 95% CI 1-6, respectively). Bronchiectasis were more common in adults (36%, 95% CI 27-46) than in children (16%, 95% CI 9-25). An overview of all reported clinical manifestations in children and adults is included in **Supplementary Table 6**.

## Discussion

To our knowledge, our study is the first systematic review and meta-analysis of pooled clinical manifestations in patients with CVID. Our findings can help clinicians to recognize CVID, and to estimate how common a clinical manifestation is in pediatric and adult CVID. We identified 134 different *presenting* clinical manifestations in patients diagnosed with CVID (the limited number of data impeded splitting up between children and adults). In addition, we identified 270 different clinical manifestations occurring during the entire course of the disease (147 in children and 170 in adults). Most frequent presenting manifestations were recurrent upper respiratory tract infections (73%), lower respiratory tract infections (73%), sinusitis (59%), pneumonia (57%), bronchitis (57%) and otitis (39%), concurrent with the first two ESID and first three JMF warning signs for PID (20, 21). However, these manifestations are also frequent in the general population and may lack discriminating value, unless their unusually recurrent and persistent nature is recognized (85). Other alerts to potential PID that are in line with the JMF warning signs that may be more discriminatory include severe bacterial infections (osteomyelitis in 4%, meningitis in 6%, septicemia in 8%, mastoiditis in 8%), which are clearly more frequent than lifetime prevalence in the general population. Recent studies already demonstrated a high incidence of antibody deficiency in patients with pneumococcal meningitis (86–88), confirming our finding of a high frequency of infectious meningitis in CVID, but we also show a high incidence of bloodstream infections, mastoiditis and to a somewhat lesser extent osteomyelitis in CVID patients. This suggests that the incidence of CVID may also be increased in patients with bloodstream infections, mastoiditis and osteomyelitis without other clear predispositions and suggest screening for CVID could be useful in these patients. This finding warrants further exploration.

One of the most reliable alerts to potential CVID was CVID in the family (12% of the total reviewed population). Both the six ESID and ten JMF warning signs make no mention of other presenting manifestations than frequent and/or severe infections, such as bronchiectasis (28%), lymphadenopathy (27%), splenomegaly (13%), chronic diarrhea (21%), inflammatory bowel disease (11%), or autoimmune hematological manifestations (autoimmune cytopenia (10%) and idiopathic thrombocytopenia (6%)). Our results suggest that also lymphoproliferative, gastrointestinal and autoimmune manifestations should be included in warning signs for predicting PID. This is important, because we still fail to detect the disease early enough. Increasing awareness of this varied and complex presentation of CVID can lead to earlier detection and initiation of treatment.

Our findings show a male predominance in children with CVID (62%), but a female predominance in adults (58%). This is also observed in atopic disease and we previously described this in patients with unclassified primary antibody deficiency (89), but it has not previously been recognized in CVID. This sex shift may indicate that etiology differs in different age groups. Early childhood male predominance suggests X-linked heredity is present in some boys diagnosed with the disease; adult female predominance suggests sex hormone effects, environmental exposure, and epigenetic influences may play a role (90). This implicates that future studies that attempt to define mechanisms that underpin CVID should be stratified according to sex.

There were clear differences in clinical manifestations occurring during the disease course between children and adults with CVID. Overall prevalence of bronchiectasis was 36% in adults vs 16% in children. Persistence of an ‘infection-only’ phenotype was much more prevalent in pediatric than in adult CVID (76 vs 38%). During childhood, three quarters of patients developed no other complications besides infections, while this percentage was much lower during follow up in adults (38%). Immune-dysregulation features, such as granulomatous-lymphocytic interstitial lung disease (15 vs 6%), chronic diarrhea (34 vs 17%), and inflammatory bowel disease (18 vs 3%) were more prominent in adults compared to children. A possible explanation could be longer ongoing inflammation and longer follow-up in the adults (43). One is an adult for many more years than one is a child, thus there are many more physician visits in the adult years and more opportunities for CVID complications to be observed. The different signs and symptoms observed in CVID between pediatric and adult age, with more non-infectious disease complications in adults, suggest that different monitoring strategies for children and adults during follow-up may be warranted.

Most common non-infectious manifestations included bronchiectasis (32%), lymphadenopathy (30%), splenomegaly (29%), polyclonal lymphocytic infiltration (29%), and autoimmune manifestations (27%). While only a quarter of CVID patients had features of immune dysregulation at presentation, this increased to about half of the patients throughout the course of the disease. This suggests that these manifestations more often occur later in the disease course. It is crucial that CVID patients are monitored for the development of these complications, because some of these are difficult to treat and associated with increased mortality (4,14). The coincidence of immunodeficiency and immune dysregulation can be explained by several mechanisms. Immunodeficiency may result in insufficient clearance of microbial antigens, and the resulting persistent antigenic exposure could then trigger granulomatous disease and autoimmunity (91). Both complications have been linked to hyperplastic germinal centers enriched with polyclonal/self-reactive B-cell clones (92), and immature B cell development (25) in CVID. In addition, low numbers of regulatory T-cells (91, 93), and an increasing number of genetic defects (94) have been associated with immune dysregulation in CVID. Additional factors, such as commensal microbial dysbiosis and epigenetic modifications remain to be better elucidated (95).

Interestingly, we found high pooled prevalences of atopic diseases both at presentation of CVID and during the entire disease course (asthma 31 vs 25%, allergic rhinitis 36 vs 18%). The pooled prevalences of asthma and allergic rhinitis are higher in CVID compared to the estimated lifetime prevalences in the general population of 13.6% (96) and 6.6% (97), respectively. This should be interpreted with caution because of the considerable heterogeneity between the studies (I^2^ >80). Also, not all patients underwent cutaneous or *in vitro* testing or spirometry to support these diagnoses, nor was it reported how often the asthma was atopic in nature. It is possible that symptoms derived from the deficient immune system were interpreted as atopic disease, on the other hand, atopic disorders could actually be more prevalent in CVID. Overlap between the symptoms of atopic diseases and immunodeficiency may lead to delayed diagnosis, so it is important to consider CVID in patients with atopic diagnoses who are insufficiently responsive to standard treatment and who also have infections. To further elucidate the association between atopic diseases and CVID, a prospective multi-center study in a large unselected CVID cohort would be needed.

A substantial number of patients developed malignancies during the disease course (10%, 95% CI 7-14). This pooled prevalence is comparable with the result of a previous focused meta-analysis of malignancy prevalence in CVID (8.6%, 95% CI 7.1-10) (98). Also, in alignment with previous reports the most common malignancies were lymphoid malignancies (5%, 95% CI 3-7) and gastric cancers (2%, 95% CI 1-4) (49, 72, 98–100). The lack of data on controls impedes comparison of our results to the normative population, but the prevalences are higher compared to the lifetime prevalence estimates in the United States population (lymphoid malignancies 2.3%, gastric cancers 0.8%) (101). The prevalence of cases with lung-, colorectal-, uterine-, liver-, and pancreatic cancer and leukemia were similar to what one might expect in the general population according to the lifetime risk statistics based on the United States population (**Supplementary Table 4**) (101).

### Strengths and Limitations

This analysis collates data from >8000 patients (850 children, 2998 adults, 4673 not specified/both) in 51 studies from 18 different countries. The included studies were conducted in Europe, North- and South America, and Asia; there were no studies from Australia or Africa. Our review adhered to rigorous methods, including a systematic search strategy, and explicit inclusion criteria (102). The findings therefore present the most comprehensive and internationally relevant presenting manifestations for clinicians worldwide.

The study has some limitations and potential sources of bias. The main limitations reflect deficits in the design and reporting of the included studies. Accuracy of our systematic analysis depends on the quality of the published and supplementary data that we included. All studies provided data on cases only, and not on controls. Therefore, we were unable to compare the frequency of clinical manifestations in CVID patients to the frequency in the general population. Publication bias could have led to overrepresentation of more complex cases of CVID, and therefore higher incidences of non-infectious complications. We did not include unpublished data. Heterogeneity between included studies was high. Most included studies provided little motivation for the selection of the clinical manifestations studied, thus it is difficult to account with certainty for the variation in number and choice of the selected clinical manifestations. The variation in reported clinical phenotypes and complications between cohorts may stem from differences in study populations (for instance, due to access to health care, rate at which patients are properly diagnosed, degree of consanguinity, or population genetic differences), use of different methods to diagnose findings, underreporting of histological diagnoses because biopsies are not performed, and the use of different definitions for CVID. Full consensus regarding the definition of CVID does not yet exist (103). Also, in a few series, a small proportion of pediatric and adult patients had opportunistic infections and/or a low CD4 T-cell count, and those patients should actually be classified as a combined immunodeficiency. This phenotype has been re-named in adults as late onset combined immunodeficiency (LOCID) by the IUIS. In addition, given the rapid progress in next-generation sequencing, in non-consanguineous populations, a causative mutation may currently be identified in ~25% of CVID patients (104). Ideally, patients with monogenic diseases and LOCID should have been excluded from our analysis, but it was not possible to identify them exactly in the described cohorts (105). Transient hypogammaglobulinemia of infancy may have been misdiagnosed as CVID in some of the children included in the different series. As we only included children diagnosed at age >4 years it is unlikely this accounts for a large percentage of included children.

### Implications for Future Research

Our study identified two key limitations in the current evidence base on CVID presentation. First, we found relatively few studies that explicitly reported data on clinical signs and symptoms at or before diagnosis of the disease. We lack data on the frequency and time of onset of symptoms from the first symptoms at home to the final diagnosis. Second, only few studies compared pediatric with adult CVID. Further large, multicenter, prospective cohort studies, separately describing children and adults, would address these gaps.

## Conclusions

In conclusion, this meta-analysis confirms the high frequency of upper and/or lower respiratory tract infections in CVID at presentation, but also shows a remarkably high incidence of severe bacterial infections (osteomyelitis in 4%, meningitis in 6%, septicemia in 8%, mastoiditis in 8%) compared to lifetime prevalence in the general population. This suggests that the incidence of CVID may also be high in patients with severe bacterial infections without other clear predispositions and suggests screening for CVID might be useful in these patients. These findings warrant further exploration. In addition, CVID patients commonly present with other manifestations than frequent or severe infections – which are not included in ESID and JMF warning signs for identifying patients with primary immunodeficiencies. Not only the infectious, but also the immune dysregulation features (shown in **Figure 2**), should alert to the possibility of CVID, regardless whether they occur with or without recurrent infections. The bimodal sex distribution in patients with CVID implicates that future studies that attempt to define mechanisms that underpin CVID should be stratified according to sex.

## Data Availability Statement

The original contributions presented in the study are included in the article/supplementary material. Further inquiries can be directed to the corresponding author.

## Author Contributions

LJ, MF, and EV devised the study. LJ, MF, and EV acquired the data. LJ carried out data analysis and drafted the manuscript. All authors contributed to the article and approved the submitted version.

## Conflict of Interest

MF received research support to study innovative antibody preparations from CSL Behring outside the submitted work. EV received an unrestricted research grant for the unPAD study outside the submitted work from Shire/Takeda.

The remaining author declares that the research was conducted in the absence of any commercial or financial relationships that could be construed as a potential conflict of interest.

## Abbreviations

CASP, critical appraisal skills program; CVID, common variable immunodeficiency disorders; ESID, European Society of Immunodeficiency; ICON, international consensus document; LOCID, late onset combined immunodeficiency; MOOSE, meta-analysis of observational studies in epidemiology; PID, primary immunodeficiency; SE, standard error; STROBE, strengthening the reporting of observational studies in epidemiology.
