# Supplementary material for: Lessons Learned From the Clinical Presentation of Common Variable Immunodeficiency Disorders: A Systematic Review and Meta-Analysis
Source: Front Immunol. 2021 Mar 23;12:620709. doi: 10.3389/fimmu.2021.620709 (PMC8021796; doi:10.3389/fimmu.2021.620709)
Supplement: Supplementary file 1 [file DataSheet_1.pdf]

Supplementary Table 1. Handling overlapping data.

**Problem**	**How this was dealt with**	**Excluded studies**	**Included study**	**Studies of whom overlapping variables were excluded**	**Study of whom all variables were included**
Multiple updates of a cohort were published with a complete overlapping recruitment period.	The study with the largest dataset describing the overall clinical picture of the cohort was included.	Aghamohammadi 2006<br>Aghamohammadi 2010<br>Mokhtari 2016<br>Valizadeh 2017	Aghamohammadi 2014		
A cohort, originating from a single centre, that been extensively described and published, was later included in a registry or multicentre cohort report.	When there were overlapping variables between the single-centre and registry/multicentre-study, the overlapping variables were included only from the larger (and often more recent) multicentre study.			Piqueras 2003	Boursiquot 2013
				Mohammadinejad 2015	Aghamohammadi 2014
				Graziano 2017	Pulvirenti 2018
				Cunningham-Rundles 1999<br>Ardeniz 2009<br>Resnick 2012<br>Filion 2018<br>Baloh 2019	Feuille 2018
The same centre/ registry published an article about their total cohort and another article in which children and/or adults were separately described.	Children- and adult-specific overlapping data were only included in the subgroup analysis for children vs adults.			Mohammadinejad 2012	Aghamohammadi 2014
				Sanchez 2017<br>Farmer 2018	Feuille 2018
The study focused only on certain clinical manifestations.	-When this was the most recent update of the cohort, only the overlapping clinical	Wang 2005<br>Zhang 2007	Resnick 2012	Oksenhendler 2007<br>Boileau 2011	Boursiquot 2013

	manifestations were removed from earlier publications on the cohort(s).<br>-When these studies with a focus were followed by studies reporting a larger cohort, which described the same clinical manifestations, this focused study was excluded.			Khodadad 2007	Yazdani 2016<br>Aghamohammadi 2014
A large multicentre cohort included cohorts from centres that already were previously published as single-centre cohort in more detail.	When only few clinical manifestations were described in the multicentre cohort, this large cohort was not included in the analysis and preference was given to the smaller cohorts which were described in more detail.	Gathmann 2014			
Multicentre studies with partial overlapping included centres.	The largest multicentre study describing the overall clinical picture of the cohort was included.	Wehr 2008<br>Packwood 2010		Chapel 2008	
