# Supplementary material for: Lessons Learned From the Clinical Presentation of Common Variable Immunodeficiency Disorders: A Systematic Review and Meta-Analysis
Source: Front Immunol. 2021 Mar 23;12:620709. doi: 10.3389/fimmu.2021.620709 (PMC8021796; doi:10.3389/fimmu.2021.620709)
Supplement: Supplementary file 2 [file DataSheet_2.pdf]

Supplementary Table 2. Quality assessment of studies meeting eligibility criteria for potential inclusion.

STUDY	1.CASE DEFINITION		2. CASE SELECTION				3. DATA SELECTION			INCLUDE PAPER?
	Adequate definition?	Overall acceptable/ not	Clear baseline characteristics (eg. age and sex)	Representative cohort?	Adequate ascertainment?	Overall acceptable/ not	Standardised collection? (=use of proforma)	Objective measurement? (eg. utrasound findings)	Overall acceptable/ not	
Aghamohammadi (2006)	Y	Y	Y	N	Y	Y	Y	Unclear	Y	**N***
Aghamohammadi (2010)	Y	Y	Y	N	Y	Y	Y	Unclear	Y	**N***
Aghamohammadi (2014)	Y	Y	Y	N	Y	Y	Y	Unclear	Y	**Y**
Agondi (2010)	Unclear	N	Y	Unclear	Unclear	N	Unclear	Y	Y	**N**
Almejun (2012)	Y	Y	Y	Y	Y	Y	Y	Y	Y	**Y**
Ardeniz (2009)	Y	Y	Y	Y	N	Y	Y	Y	Y	**Y**
Ardeniz (2010)	Y	Y	Y	Y	Y	Y	Y	Y	Y	**Y**
Arshi (2016)	Y	Y	Y	Y	Y	Y	Y	Y	Y	**Y**
Aydogan (2008)	Y	Y	Y	N	N	N	Y	Y	Y	**N**
Bahceci (2019)	Y	Y	Y	Y	Y	Y	Y	Y	Y	**Y**
Baloh (2019)	Y	Y	Y	Y	Y	Y	Y	Y	Y	**Y**
Barton (2014)	Y	Y	Y	Y	Y	Y	Y	Y	Y	**Y**
Bates (2004)	Y	Y	Y	Y	Y	Y	Y	Y	Y	**Y**
Bezrodnik (2011)	Y	Y	Y	Unclear	Unclear	N	Unclear	Unclear	N	**N**
Bjelac (2018)	Y	Y	Y	Y	Y	Y	Unclear	Y	Y	**Y**
Bouileau (2011)	Y	Y	Y	Y	Y	Y	Y	Y	Y	**Y**
Boursiquot (2013)	Y	Y	Y	N	N	N	Y	Y	Y	**Y**
Chan (2014)	Y	Y	Y	Y	Y	Y	Y	Y	Y	**Y**
Chapel (2008)	Y	Y	Y	Y	Y	Y	Y	Y	Y	**Y**
Cunningham-Rundles (1999)	Y	Y	Y	Y	Y	Y	Y	Y	Y	**Y**
Danieli (2012)	Unclear	N	Y	Y	Y	Y	Unclear	Unclear	N	**N**
Dirks (2012)	Unclear	N	N	Unclear	Unclear	N	Unclear	Unclear	N	**N**
Farmer (2018)	Unclear	Y	Y	Y	Y	Y	Y	Y	Y	**Y**
Feuille (2018)	Y	Y	Y	Y	Y	Y	Y	Y	Y	**Y**

Filion (2018)	Y	Y	Y	Y	Y	Y	Y	Y	Y	**Y**
Fischer (2017)	Y	Y	N	Y	Y	Y	Y	Unclear	Y	**Y**
Gaillard (2015)	Y	Y	Y	Y	Y	Y	y	Y	Y	**Y**
Gathmann (2014)	Y	Y	Y	Y	Y	Y	y	Y	Y	**Y**
Graziano (2017)	Y	Y	Y	Y	Y	Y	Y	Y	Y	**Y**
Grossman (2010)	Unclear	N	Y	Unclear	N	N	Unclear	Unclear	N	**N**
Hilst (2002)	Y	Y	Y	Y	Y	Y	Y	Y	Y	**Y**
Jorgensen (2016)	Y	Y	Y	Y	Y	Y	Y	Y	Y	**Y**
Kainulainen (2001)	Y	Y	Y	Y	Y	Y	Y	Unclear	Y	**Y**
Karaulov (2012)	Y	Y	Y	Unclear	Y	Y	Y	Y	Y	**Y**
Kapoussouzi (2016)	N	N	Y	Y	Y	Y	Unclear	Unclear	N	**N**
Khodadad (2007)	Y	Y	Y	Y	Y	Y	Y	Y	Y	**Y**
Kokron (2004)	Y	Y	Y	Y	Y	Y	Y	Y	Y	**Y**
Llobet (2009)	Y	Y	Y	Y	Y	Y	Y	Y	Y	**Y**
Maarschalk (2014)	Y	Y	Y	Y	N	Y	Y	Y	Y	**Y**
Maglione (2015)	Unclear	N	N	Unclear	Unclear	N	Unclear	Unclear	N	**N**
Martinez Garcia (2001)	Unclear	Y	Y	Y	N	Y	Y	Y	Y	**Y**
Mohammadinejad (2012)	Y	Y	Y	N	Y	Y	Y	Y	Y	**Y**
Mohammadinejad (2015)	Y	Y	Y	Y	Y	Y	Y	Y	Y	**Y**
Mokhtari (2016)	Y	Y	Y	N	Y	Y	Y	Y	Y	**N***
Musabak (2017)	Y	Y	Y	Y	Y	Y	Y	Y	Y	**Y**
Nisha (2013)	Unclear	N	Y	Unclear	Unclear	N	Unclear	Unclear	N	**N**
Ogershok (2006)	Y	Y	Y	Y	Y	Y	Y	Y	Y	**Y**
Ogershok (2015)	N	N	N	Unclear	Unclear	N	Unclear	Unclear	N	**N**
Oksenhendler (2007)	Y	Y	Y	Y	Y	Y	Y	Y	Y	**Y**
Oksenhendler (2012)	Unclear	N	Y	Y	Y	Y	Unclear	Unclear	N	**N**
Packwood (2010)	Y	Y	N	Y	Y	Y	Y	Y	Y	**Y**
Pereira (2009)	Unclear	N	Y	Y	Y	Y	Unclear	Unclear	N	**N**

Piatosa (2013)	Y	Y	Y	Y	Y	Y	Y	Y	Y	Y
Piqueras (2003)	Y	Y	Y	Y	Y	Y	Y	Y	Y	Y
Prokofjeva (2012)	Unclear	N	N	Unclear	Unclear	N	Unclear	Unclear	N	N
Pulvirenti (2018)	Y	Y	Y	Y	Y	Y	Y	Y	Y	Y
Quinti (2007)	Y	Y	Y	Y	Y	Y	Y	Y	Y	Y
Ramirez-Vargas (2012)	Y	Y	Y	Y	Y	Y	Y	Y	Y	Y
Resnick (2011)	Y	Y	Y	Y	Y	Y	Y	Y	Y	Y
Sanchez (2017)	Y	Y	Y	Y	Y	Y	Y	Y	Y	Y
Santaella (2005)	Unclear	Y	Y	Y	Y	Y	Y	Y	Y	Y
Seba (2012)	Unclear	Y	Y	Unclear	Unclear	N	Unclear	Unclear	N	N
Selenius (2017)	Y	Y	Y	Y	Y	Y	Y	Y	Y	Y
Seminario (2017)	Unclear	N	Y	Y	Y	Y	Unclear	Unclear	N	N
Sperlich (2017)	Y	Y	Y	Y	Y	Y	Y	Y	Y	Y
Tampella (2009)	Unclear	N	N	Unclear	Y	N	Unclear	Unclear	N	N
Thickett (2002)	Y	Y	Y	Y	Y	Y	Y	Y	Y	Y
Urschel (2009)	Y	Y	Y	Y	Y	Y	Y	Y	Y	Y
Ven (2009)	Y	Y	Y	Y	Y	Y	Y	Y	Y	Y
Verma (2013)	Unclear	N	Y	Y	Y	Y	Unclear	Unclear	N	N
Wang (2005)	Y	Y	Y	Y	Y	Y	Y	Y	Y	N**
Wehr (2008)	Y	Y	Y	Y	Y	Y	Y	Y	Y	Y
Westh (2017)	N	N	Y	Y	Y	Y	Y	Y	Y	N
Więsik-Szewczyk (2018)	Y	Y	Y	Y	Y	Y	Y	Y	Y	Y
Yazdani (2016)	Y	Y	Y	Y	Y	Y	Y	Y	Y	Y
Yong (2010)	Y	Y	Y	Y	Y	Y	Y	Y	Y	Y
Yonker (2012)	Unclear	N	Y	Unclear	Unclear	N	Unclear	Unclear	N	N
Valizadeh (2017)	Y	Y	Y	N	Y	N	Y	Y	Y	N*
Zhang (2007)	Y	Y	Y	Y	Y	Y	Y	Y	Y	N**

*The same study population as reported in the published article by Aghamohammadi in 2014 is described.

**This study focused only on certain clinical manifestations and was followed by a study reporting a larger cohort of the same center, which described the same clinical manifestations (Resnick et al. 2012).
