# Supplementary material for: Lessons Learned From the Clinical Presentation of Common Variable Immunodeficiency Disorders: A Systematic Review and Meta-Analysis
Source: Front Immunol. 2021 Mar 23;12:620709. doi: 10.3389/fimmu.2021.620709 (PMC8021796; doi:10.3389/fimmu.2021.620709)
Supplement: Supplementary file 3 [file DataSheet_3.pdf]

Supplementary Table 3. Definitions of common variable immunodeficiency.

ESID/PAGID	ESID diagnostic criteria	IUIS criteria	WHO scientific group	ICON
Probable<br>Marked decrease (≥2 SD below mean for age) in serum IgG and IgA and:<br>1.Onset of immunodeficiency ≥2 years of age<br>2.Absent isohemagglutinins and/or poor response to vaccines<br>3.Defined causes of hypogammaglobulinemia have been excluded<br><br>Possible<br>Marked decrease (≥2 SD below mean for age) in one of the major isotypes (IgM, IgG, and IgA) and:<br>1.Onset of immunodeficiency ≥2 years of age<br>2.Absent isohemagglutinins and/or poor response to vaccines<br>3.Defined causes of hypogammaglobulinemia have been excluded	At least one of the following:<br>-increased susceptibility to infection<br>-autoimmune manifestations<br>-granulomatous disease<br>-unexplained polyclonal lymphoproliferation<br>-affected family member with antibody deficiency<br><br>AND marked decrease of IgG and IgA with or without low IgM levels;<br><br>*AND* at least one of the following:<br>-poor antibody response to vaccines<br>-low switched memory B cells (<70% of age-related normal value)<br><br>AND secondary causes have been excluded (e.g., infection, protein loss, medication, malignancy)	Decreased serum levels of IgG, IgA, and/or IgM (≥2 SD below mean for age)	Decreased serum levels of IgG, IgA, and/or IgM (≥2 SD below mean for age)	Decreased serum levels of IgG, IgA, and/or IgM (≥2 SD below mean for age), impairment of specific antibody responses, and, occasionally, reductions in B-cell numbers

Abbreviations: ESID, European Society for Immunodeficiencies; ICON, International Consensus Document; IUIS, International Union of Immunological Societies; PAGID, Pan-American Group for Immunodeficiency; WHO, World Health Organization.
