# Supplementary material for: Lessons Learned From the Clinical Presentation of Common Variable Immunodeficiency Disorders: A Systematic Review and Meta-Analysis
Source: Front Immunol. 2021 Mar 23;12:620709. doi: 10.3389/fimmu.2021.620709 (PMC8021796; doi:10.3389/fimmu.2021.620709)
Supplement: Supplementary file 4 [file DataSheet_4.pdf]

Supplementary Table 4. Subgroup analyses of clinical manifestations at presentation vs overall clinical manifestations during the disease course compared to population lifetime prevalence.

	At or before diagnosis		Overall		General population prevalence
**Manifestation**	**Pooled proportion (95% CI)**	**I^2**	**Pooled proportion (95% CI)**	**I^2**	**Lifetime prevalence percentage estimates from various sources**
**Clinical phenotype[a]**					
Infections only	93 (78-100)	74	48 (39-58)	93	n/a
Autoimmunity	19 (11-28)	81	27 (22-32)	91	3.9% (1)
Polyclonal lymphocytic infiltration	n/a	n/a	29 (22-37)	92	<<1%
Enteropathy	n/a	n/a	9 (6-13)	86	<<1%
Lymphoid malignancy	2 (0-4)	0	5 (3-7)	81	2.1 + 0.2 = 2.3% (2)
Malignancy	4 (1-8)	0	10 (7-14)	87	39.5 (2)
**Respiratory involvement**					
Otitis	39 (28-49)	88	43 (35-51)	86	n/a[b]
Sinusitis	59 (47-71)	93	67 (57-77)	95	11.2% (3)
Upper respiratory tract infections	73 (53-89)	96	84 (75-92)	89	n/a[b]
Pneumonia	57 (43-69)[c]	94	62 (54-70)[d]	92	n/a[b]
Bronchitis	57 (42-72)	92	62 (44-78)	97	n/a[b]
Lower respiratory tract infections	73 (56-86)	85	78 (64-90)	84	n/a
Bronchiectasis	28 (18-40)	91	32 (26-38)	93	0.27% (4)
Lymphocytic interstitial pneumonia	n/a	n/a	3 (1-5)	40	n/a
GLILD	n/a	n/a	15 (7-25)	96	n/a
Lymphoid hyperplasia[e]	n/a	n/a	10 (5-17)	91	n/a
Asthma	31 (3-69)	92	25 (17-35)	94	13.6% (3)
Allergic rhinitis	36 (9-68)	82	18 (8-31)	95	6.6% (5)
Idiopathic lung fibrosis	n/a	n/a	12 (1-30)	92	0.01% (6)
Emphysema	4 (1-10)	0	4 (1-10)	0	1.3% (3)
Pulmonary lobe resection	5 (3-8)	0	5 (3-8)	0	n/a
Respiratory tract infections	86 (62-99)	94	92 (78-99)	93	n/a
Lung cancer	n/a	n/a	1 (0-1)	44	6.3% (2)
Allergy	16 (0-63)	96	33 (13-56)	94	4.6 (7)
Mastoiditis	8 (3-16)	0	3 (1-7)	64	<0.1% (1.2/10,000 child years) (8)
Interstitial lung disease	n/a	n/a	13 (6-23)	88	n/a
Lung nodules	n/a	n/a	3 (1-6)	53	n/a
Pharyngitis/tonsillitis	n/a	n/a	18 (12-24)	68	n/a[b]
Conjunctivitis	14 (6-25)	73	10 (5-16)	87	n/a[b]
COPD	n/a	n/a	34 (14-58)	61	1.7% (9)
**Gastrointestinal involvement**					
Chronic diarrhea of unknown origin	21 (11-34)	92	27 (21-34)	88	4-5% (10)
Gastrointestinal tract infection	29 (15-44)[f]	72	29 (21-38)[g]	92	n/a[b]
Intestinal granulomatosis	n/a	n/a	1 (0-4)	81	<<0.01%
Inflammatory bowel disease	11 (3-23)	59	10 (6-16)	90	0.71% (1)
Crohn's disease	n/a	n/a	3 (2-4)	0	0.23% (1)
Ulcerative colitis	n/a	n/a	2 (1-3)	0	0.48% (1)
Lymphocytic colitis	n/a	n/a	9 (4-16)	0	n/a

Eosinophilic inflammation	n/a	n/a	5 (2-10)	0	<<1% (11)
Celiac disease	1 (0-4)	23	3 (2-4)	25	0.06% (1)
Villous atrophy[h]	n/a	n/a	11 (1-28)	83	n/a
Atrophic gastritis	n/a	n/a	5 (3-9)	62	n/a (12)
Protein losing enteropathy	n/a	n/a	1 (0-3)	0	<<1% (13)
Stomach cancer	n/a	n/a	2 (1-4)	61	0.8% (2)
Colorectal cancer	n/a	n/a	1 (0-2)	40	4.2% (2)
Gastro-esophageal reflux disease	n/a	n/a	16 (8-25)	84	22.2% (14)
Gastritis	n/a	n/a	28 (22-35)	17	n/a
Peritonitis	3 (1-8)	0	4 (1-8)	0	<<1%
Malabsorption	n/a	n/a	13 (4-26)	96	n/a
**Glandular tissue**					
Sjogren's syndrome	13 (2-25)	67	3 (1-6)	86	0.06% (1)
Breast cancer	n/a	n/a	1 (1-2)	39	12.9% (2)
Cervix cancer	n/a	n/a	1 (1-2)	0	0.6% (2)
Uterine cancer	n/a	n/a	1 (0-2)	28	3.1% (2)
**Hepatic involvement**					
Autoimmune hepatitis	n/a	n/a	4 (0-10)	89	0.045% (1)
Primary biliary cirrhosis	n/a	n/a	2 (1-3)	39	<<1%
Unexplained hepatomegaly	n/a	n/a	14 (8-72)	93	<<1%
Viral hepatitis	n/a	n/a	4 (2-6)	64	n/a
Liver granuloma	n/a	n/a	3 (1-6)	68	n/a
Liver cirrhosis	n/a	n/a	4 (1-9)	0	n/a
Liver cancer	n/a	n/a	1 (0-1)	44	1% (2)
Hepatitis (not further specified)	n/a	n/a	4 (0-12)	94	n/a
**Urinary tract involvement**					
Urinary tract infection	14 (5-25)	83	14 (11-18)	66	30% (female 50%, male 12%) (15)
Pyelonephritis	n/a	n/a	8 (0-22)	77	2% (15)
**Hematologic involvement**					
Idiopathic thrombocytopenia	6 (4-9)	20	10 (7-14)	84	0.045% (1)
Autoimmune cytopenia	10 (7-14)	0	13 (10-17)	86	n/a
Autoimmune hemolytic anemia	3 (2-5)	0	6 (4-8)	77	0.013% (1)
Autoimmune neutropenia	3 (0-9)	48	4 (2-6)	68	<<0.1% (16)
Unknown/other neutropenia	n/a	n/a	6 (3-10)	86	<<0.1% (16)
Evans syndrome	n/a	n/a	2 (1-2)	0	<<0.1% (17)
Iron deficiency anemia	n/a	n/a	10 (2-22)	97	12.2% (18)[i]
Pernicious anemia	n/a	n/a	3 (1-5)	84	0.05% (1)
Bloodstream infection	8 (4-13)	77	9 (5-13)	85	1.2% (19)
**Neurological involvement**					
Headache/migraine	n/a	n/a	17 (n/a)	n/a	15.9% (20)
Infectious meningitis	5 (3-9)[j]	76	7 (5-9)[k]	62	0.3% (21)
Myasthenia gravis	n/a	n/a	0 (0-1)	47	0.018% (1)
Multiple sclerosis	n/a	n/a	0 (0-1)	22	0.19% (1)
Bell's palsy	n/a	n/a	1 (0-2)	52	0.6% (22)
Polyneuropathy	n/a	n/a	3 (1-6)	64	1% (23)
Epilepsy	n/a	n/a	6 (2-11)	71	10% (24)
**Bone and joint involvement**					

Septic arthritis	2 (1-4)	0	4 (1-8)	72	0.15% (25)
Rheumatoid arthritis	4 (1-10)	70	3 (2-4)	42	0.5% (1)
Seronegative arthritis	6 (2-12)	21	4 (2-7)	74	21.4% (26)
Juvenile idiopathic arthritis	n/a	n/a	1 (0-4)	82	0.073% (1)
Psoriatic arthritis	n/a	n/a	0 (0-1)	47	n/a
Osteomyelitis	4 (1-9)	16	4 (1-8)	83	1.7% (27)
Multiple myeloma	n/a	n/a	0 (0-1)	0	n/a
Kaposi's sarcoma	n/a	n/a	0 (0-1)	0	n/a
Osteoporosis/osteopenia	n/a	n/a	10 (1-25)	92	27% (28)
**Lymph node and spleen involvement**					
Lymphadenopathy	27 (0-90)	96	30 (20-42)	96	64% (29)
Non-malignant lymphoproliferation	n/a	n/a	28 (16-40)	94	n/a
Splenomegaly	13 (5-23)	90	29 (22-37)	94	2% (30)
Non-hodgkin's lymphoma	n/a	n/a	4 (3-5)	56	2.1% (2)
Splenectomy	3 (1-6)	0	6 (3-8)	57	n/a
Pancreatic cancer	n/a	n/a	2 (0-7)	57	1.6% (2)
Leukemia	n/a	n/a	1 (1-2)	22	1.5% (2)
Granuloma in lymph node	n/a	n/a	2 (0-5)	80	n/a
Granuloma in spleen	n/a	n/a	1 (0-2)	24	n/a
Granuloma in bone marrow	n/a	n/a	0 (0-1)	0	n/a
**Endocrinological involvement**					
Thyroid disease (all)	16 (0-43)	92	9 (4-16)	95	12% (31)
Autoimmune thyroiditis	3 (0-8)	68	4 (2-6)	80	0.058% (1)
Diabetes mellitus	2 (0-7)	0	2 (1-3)	18	0.9% (type 1) (1)
Thyroid cancer	n/a	n/a	1 (0-3)	68	1.3% (2)
**Muco/cutaneous involvement**					
Psoriasis	n/a	n/a	3 (2-5)	38	0.32% (1)
Atopic dermatitis	12 (4-24)	0	12 (7-18)	86	2.1-4.9% (32)
Alopecia	n/a	n/a	3 (2-4)	0	0.031% (1)
Vitiligo	2 (1-5)	0	5 (3-8)	81	0.022% (1)
Warts	n/a	n/a	5 (4-7)	0	n/a
Skin cancer	n/a	n/a	3 (1-5)	70	20% (33)
Skin infections	10 (6-15)	43	13 (10-17)	59	n/a
Cutaneous abscesses	3 (1-6)	17	5 (2-10)	79	n/a
(Recurrent) herpes zoster	n/a	n/a	9 (5-14)	84	23.8% (34)
(Recurrent) herpes simplex	n/a	n/a	9 (2-10)	89	38.3% (35)
Oral candidiasis	3 (1-5)	0	6 (2-11)	91	n/a
Genital candidiasis	n/a	n/a	6 (3-12)	0	30-50% (36)
Urticaria	n/a	n/a	5 (3-8)	71	11.5% (37)
Oral/dental infections	10 (3-21)	0	4 (3-6)	5	n/a
Skin granuloma	n/a	n/a	1 (0-2)	41	n/a
Severe varicella	n/a	n/a	4 (2-7)	0	n/a
Recurrent parotitis	n/a	n/a	6 (0-3)	93	n/a
Clubbing	n/a	n/a	19 (0-73)	97	n/a
Aphthous lesions	n/a	n/a	16 (5-31)	77	0.1-1.2% (38)
**Other clinical manifestations**					
Growth retardation/weight loss	8 (1-22)	82	13 (7-22)	87	n/a
Unexplained granuloma	5 (4-7)	0	12 (9-16)	79	n/a
Vasculitis	n/a	n/a	2 (1-3)	44	n/a

Systemic lupus erythematodus	2 (0-6)	28	1 (1-2)	48	0.048% (1)
Asymptomatic	6 (2-11)	74	n/a	n/a	n/a

[a]According to Chapel et al. (39)

[b]The lifetime prevalence of respiratory and gastrointestinal tract infections is also high in the general population and may lack any discriminating value, unless their unusually recurrent and persistent nature is recognized. Because of the absence of data on the number of infectious episodes in the included studies, population lifetime prevalence percentages are not shown.

[c]Streptococcus pneumoniae 11% (6-19), Haemophilus influenzae 17% (0-49), mycobacterial infection 5% (2-8).

[d]Streptococcus pneumonia 15% (8-23), Haemophilus influenzae 19% (8-33), Moraxella Catharalis 7% (0-19), Staphylococcus aureus 7% (3-12), Mycobacterial infection 1% (0-2), Pneumocystis Jiroveci 1% (0-2), Pseudomonas 6% (2-10), Aspergillus 3% (1-5), Enterobacteriaceae 6% (2-13), Mycoplasma 2% (0-4).

[e]Not only pulmonary.

[f]Giardia intestintalis 11% (3-22), Salmonella species 6% (3-11).

[g]Giardia intestinalis 13% (7-21), Salmonella species 6% (2-12), Campylobacter species 4% (1-8), Clostridium difficile 2% (1-3), cytomegalovirus 2% (0-7), Cryptosporidium species 1% (0-2), Helicobacter pylori 9% (3-18), Escherichia coli 8% (4-13), Candida species 10% (4-19), Strongyloidiasis 7% (3-13).

[h]Histologic findings of villous atrophy are similar to those found in patients with celiac disease, but with some differences; in CVID plasma cells are absent from the intestinal lamina propria, and the crypt epithelium is not hyperplastic.

[i]Prevalence, not lifetime prevalence.

[j]Streptococcus pneumonia 3% (2-6), Haemophilus influenzae 1% (0-3).

[k]Streptococcus pneumonia 3% (2-6), Haemophilus influenzae 1% (0-3).

Abbreviations: COPD, chronic obstructive pulmonary disease; GLILD, Granulomatous-lymphocytic interstitial lung disease; n/a, not applicable.

References

1. Eaton WW, Pedersen MG, Atladóttir HO, Gregory PE, Rose NR, Mortensen PB. The prevalence of 30 ICD-10 autoimmune diseases in Denmark. Immunol Res. 2010 Jul;47(1–3):228–31.
2. https://seer.cancer.gov/statfacts/html/all.html.
3. https://ftp.cdc.gov/pub/Health_Statistics/NCHS/NHIS/SHS/2018_SHS_Table_A-2.pdf.
4. https://www.erswhitebook.org/chapters/bronchiectasis/.
5. Ghouri N, Hippisley-Cox J, Newton J, Sheikh A. Trends in the epidemiology and prescribing of medication for allergic rhinitis in England. J R Soc Med. 2008 Sep;101(9):466–72.
6. Ley B, Collard HR. Epidemiology of idiopathic pulmonary fibrosis. Clin Epidemiol. 2013 Nov;5:483–92.
7. Simpson CR, Newton J, Hippisley-Cox J, Sheikh A. Incidence and prevalence of multiple allergic disorders recorded in a national primary care database. J R Soc Med. 2008 Nov;101(11):558–63.
8. Thompson PL, Gilbert RE, Long PF, Saxena S, Sharland M, Wong ICK. Effect of antibiotics for otitis media on mastoiditis in children: a retrospective cohort study using the United kingdom general practice research database. Pediatrics. 2009 Feb;123(2):424–30.
9. Simpson CR, Hippisley-Cox J, Sheikh A. Trends in the epidemiology of chronic obstructive pulmonary disease in England: a national study of 51 804 patients. Br J Gen Pract J R Coll Gen Pract. 2010 Jul;60(576):277–84.
10. Arasaradnam RP, Brown S, Forbes A, Fox MR, Hungin P, Kelman L, et al. Guidelines for the investigation of chronic diarrhoea in adults: British Society of Gastroenterology, 3rd edition. Gut. 2018 Aug;67(8):1380–99.
11. Shaheen NJ, Mukkada V, Eichinger CS, Schofield H, Todorova L, Falk GW. Natural history of eosinophilic esophagitis: a systematic review of epidemiology and disease course. Dis esophagus Off J Int Soc Dis Esophagus. 2018 Aug;31(8).
12. Rodriguez-Castro KI, Franceschi M, Noto A, Miraglia C, Nouvenne A, Leandro G, et al. Clinical manifestations of chronic atrophic gastritis. Acta Biomed. 2018 Dec;89(8-S):88–92.
13. Nagra N, Dang S. Protein Losing Enteropathy. In Treasure Island (FL); 2020.
14. Wang H-Y, Leena KB, Plymoth A, Hergens M-P, Yin L, Shenoy KT, et al. Prevalence of gastro-esophageal reflux disease and its risk factors in a community-based population in southern India. BMC Gastroenterol. 2016 Mar;16:36.
15. Foxman B. Epidemiology of urinary tract infections: incidence, morbidity, and economic costs. Dis Mon. 2003 Feb;49(2):53–70.
16. Farruggia P, Dufour C. Diagnosis and management of primary autoimmune neutropenia in children: insights for clinicians. Ther Adv Hematol. 2015 Feb;6(1):15–24.
17. Jaime-Pérez JC, Aguilar-Calderón PE, Salazar-Cavazos L, Gómez-Almaguer D. Evans syndrome: clinical perspectives, biological insights and treatment modalities. J Blood Med. 2018;9:171–84.
18. Lopez A, Cacoub P, Macdougall IC, Peyrin-Biroulet L. Iron deficiency anaemia. Lancet (London, England). 2016 Feb;387(10021):907–16.
19. Laupland KB, Gregson DB, Zygun DA, Doig CJ, Mortis G, Church DL. Severe bloodstream infections: a population-based assessment. Crit Care Med. 2004 Apr;32(4):992–7.
20. https://ftp.cdc.gov/pub/Health_Statistics/NCHS/NHIS/SHS/2018_SHS_Table_A-5.pdf.
21. McGill F, Griffiths MJ, Bonnett LJ, Geretti AM, Michael BD, Beeching NJ, et al. Incidence, aetiology, and sequelae of viral meningitis in UK adults: a multicentre prospective observational cohort study. Lancet Infect Dis. 2018 Sep;18(9):992–1003.
22. Savettieri G, Salemi G, Rocca WA, Meneghini F, Santangelo R, Morgante L, et al. Incidence and lifetime prevalence of Bell’s palsy in two Sicilian municipalities. Sicilian Neuro-Epidemiologic Study (SNES) Group. Acta Neurol Scand. 1996 Jul;94(1):71–5.
23. Hanewinckel R, van Oijen M, Ikram MA, van Doorn PA. The epidemiology and risk factors of chronic polyneuropathy. Eur J Epidemiol. 2016 Jan;31(1):5–20.
24. https://www.who.int/news-room/fact-sheets/detail/epilepsy.
25. McBride S, Mowbray J, Caughey W, Wong E, Luey C, Siddiqui A, et al. Epidemiology, Management, and Outcomes of Large and Small Native Joint Septic Arthritis in Adults. Clin Infect Dis an Off Publ Infect Dis Soc Am. 2020 Jan;70(2):271–9.
26. https://ftp.cdc.gov/pub/Health_Statistics/NCHS/NHIS/SHS/2018_SHS_Table_A-4.pdf.
27. Kremers HM, Nwojo ME, Ransom JE, Wood-Wentz CM, Melton LJ 3rd, Huddleston PM 3rd. Trends in the epidemiology of osteomyelitis: a population-based study, 1969 to 2009. J Bone Joint Surg Am. 2015 May;97(10):837–45.
28. https://www.iofbonehealth.org/facts-statistics.

29. https://www-uptodate-com.ru.idm.oclc.org/contents/peripheral-lymphadenopathy-in-children-etiology?search=Peripheral%20lymphadenopathy%20in%20children:%20Etiology&source=search_result&selectedTitle=1~150&usage_type=default&display_rank=1.
30. Chapman J, Bansal P, Goyal A, Azevedo AM. Splenomegaly. In Treasure Island (FL); 2020.
31. https://www.thyroid.org/media-main/press-room/.
32. Barbarot S, Auziere S, Gadkari A, Girolomoni G, Puig L, Simpson EL, et al. Epidemiology of atopic dermatitis in adults: Results from an international survey. Allergy. 2018 Jun;73(6):1284–93.
33. https://www.skincancer.org.
34. Bowsher D. The lifetime occurrence of Herpes zoster and prevalence of post-herpetic neuralgia: A retrospective survey in an elderly population. Eur J Pain. 1999 Dec;3(4):335–42.
35. Malvy D, Ezzedine K, Lançon F, Halioua B, Rezvani A, Bertrais S, et al. Epidemiology of orofacial herpes simplex virus infections in the general population in France: results of the HERPIMAX study. J Eur Acad Dermatol Venereol. 2007 Nov;21(10):1398–403.
36. Jacob L, John M, Kalder M, Kostev K. Prevalence of vulvovaginal candidiasis in gynecological practices in Germany: A retrospective study of 954,186 patients. Curr Med Mycol. 2018 Mar;4(1):6–11.
37. Torsten Zuberbier, Clive Grattan MM. Urticaria and Angioedema. 2010.
38. Zain RB. Oral recurrent aphthous ulcers/stomatitis: prevalence in Malaysia and an epidemiological update. J Oral Sci. 2000 Mar;42(1):15–9.
39. Chapel H, Lucas M, Lee M, Bjorkander J, Webster D, Grimbacher B, et al. Common variable immunodeficiency disorders: division into distinct clinical phenotypes. Blood. 2008 Jul;112(2):277–86.
