# Supplementary material for: Lessons Learned From the Clinical Presentation of Common Variable Immunodeficiency Disorders: A Systematic Review and Meta-Analysis
Source: Front Immunol. 2021 Mar 23;12:620709. doi: 10.3389/fimmu.2021.620709 (PMC8021796; doi:10.3389/fimmu.2021.620709)
Supplement: Supplementary file 5 [file DataSheet_5.pdf]

Supplementary Table 5. Clinical manifestations in patients with common variable immunodeficiency[a]

Manifestation	Pooled proportion (95% CI)	Nr. of participants	Nr. of studies
**Clinical phenotype[b]**			
Infections only	48 (39-58)	1677	16
Autoimmunity	27 (22-32)	4061	28
Polyclonal lymphocytic infiltration	29 (22-37)	2252	9
Enteropathy	9 (6-13)	2400	10
Lymphoid malignancy	5 (3-7)	3392	20
Malignancy	10 (7-14)	2289	25
**Respiratory involvement**			
Hypersensitivity pneumonitis	1 (n/a)	69	1
Otitis	43 (35-51)	11444	18
Sinusitis	67 (57-77)	1887	21
Upper respiratory tract infections	84 (75-92)	794	15
Pneumonia	62 (54-70)[c]	1876	22
Bronchitis	62 (44-78)	1166	8
Lower respiratory tract infections	78 (64-90)	270	8
Bronchiectasis	32 (26-38)	3720	31
Lymphocytic interstitial pneumonia	3 (1-5)	928	5
GLILD	15 (7-25)	2094	11
Lymphoid hyperplasia[d]	10 (5-17)	1667	11
Follicular bronchiolitis	1 (n/a/)	69	1
Asthma	25 (17-35)	1819	15
Allergic rhinitis	18 (8-31)	1282	7
Idiopathic lung fibrosis	12 (1-30)	247	6
Emphysema	4 (1-10)	79	2
Pulmonary lobe resection	5 (3-8)	285	6
Cryptococcal lung abscess	0 (n/a/)	248	1
Respiratory tract infections	92 (78-99)	543	5
Lung cancer	1 (0-1)	1993	4
Chronic lung disease	44 (27-63)	716	3
Allergy	33 (13-56)	419	6
Mastoiditis	3 (1-7)	532	3
Interstitial lung disease	13 (6-23)	629	4
Lung nodules	3 (1-6)	532	2
Pharyngitis/tonsillitis	18 (12-24)	575	3
Conjunctivitis	10 (5-16)	872	6
COPD	34 (14-58)	51	2
Pulmonary hypertension	3 (n/a)	988	1
Sino nasal polyps	5 (n/a)	988	1
Lung transplantations	1 (n/a)	473	1
Rhino-pharyngeal cancer	1 (n/a)	75	1
Bronchiolitis obliterans	4 (n/a)	69	1
Sarcoidosis	2 (n/a)	988	1
**Gastrointestinal involvement**			
Food intolerance	19 (n/a)	32	1
Chronic diarrhea of unknown origin	27 (21-34)	1885	14
Gastrointestinal tract infection	29 (21-38)[e]	1566	14
Intestinal granulomatosis	1 (0-4)	980	4
Inflammatory bowel disease	10 (6-16)	1940	14
Crohn's disease	3 (2-4)	703	5
Ulcerative colitis	2 (1-3)	861	7
Celiac disease	3 (2-4)	1586	9
Atrophic gastritis	5 (3-9)	652	7

Stomach cancer	2 (1-4)	1754	11
Colorectal cancer	1 (0-2)	1318	6
Eosinophilic inflammation	5 (2-10)	121	3
Protein losing enteropathy	1 (0-3)	293	2
Gastritis	28 (22-35)	286	3
Peritonitis	4 (1-8)	132	3
Villous atrophy	11 (1-28)	291	2
Malabsorption	13 (4-26)	1099	5
Autoimmune gastro-intestinal disease	13 (5-24)	1083	2
Gastro-esophageal reflux disease	16 (8-25)	565	3
Lymphocytic colitis	9 (4-16)	92	2
Chronic appendicitis	1 (n/a)	95	1
Erythema nodosum	3 (n/a)	95	1
Gastrointestinal adenocarcinoma	2 (n/a)	47	1
Primary sclerosing cholangitis	4 (n/a)	23	1
Increased intraepithelial lymphocytes	60 (n/a)	53	1
Gastric metaplasia	25 (n/a)	53	1
Stomatitis	5 (n/a)	43	1
Oesophageal cancer	0 (n/a)	473	1
Ulcerative proctitis	1 (n/a)	248	1
Sprue-like disease (intestines)	2 (n/a)	248	1
**Glandular tissue**			
Sjogren's syndrome	3 (1-6)	1736	7
Breast cancer	1 (1-2)	2079	8
Cervix cancer	1 (1-2)	1171	3
Uterine cancer	1 (0-2)	679	2
**Hepatic involvement**			
Autoimmune hepatitis	4 (0-10)	1337	5
Primary biliary cirrhosis	2 (1-3)	1011	2
Unexplained hepatomegaly	14 (8-72)	1507	8
Viral hepatitis	4 (2-6)	1194	6
Liver granuloma	3 (1-6)	1058	5
Liver cirrhosis	4 (1-9)	92	2
Liver cancer	1 (0-1)	928	2
Hepatitis (not further specified)	4 (0-12)	816	3
Liver abscess	1 (n/a)	69	1
Autoimmune liver disease	8 (n/a)	988	1
Nodular regenerative hyperplasia	1 (n/a)	988	1
**Urinary tract involvement**			
Urinary tract infection	14 (11-18)	1267	12
Pyelonephritis	8 (0-22)	205	2
Granuloma in kidney	0 (n/a)	455	1
Genitourinary cancer	3 (n/a)	455	1
Prostatic cancer	1 (n/a)	455	1
Nephrotic syndrome	1 (n/a)	248	1
**Hematologic involvement**			
Idiopathic thrombocytopenia	10 (7-14)	2717	18
Autoimmune cytopenia	13 (10-17)	3131	17
Autoimmune hemolytic anemia	6 (4-8)	2895	20
Autoimmune neutropenia	4 (2-6)	1944	9
Unknown/other neutropenia	6 (3-10)	1653	8
Evans syndrome	2 (1-2)	1208	3
Polycythaemia	0 (n/a)	224	1

Leukopenia	9 (n/a)	77	1
Haemolytic anaemia	5 (n/a)	990	1
Iron deficiency anemia	10 (2-22)	1322	2
Pernicious anemia	3 (1-5)	2142	10
Bloodstream infection	9 (5-13)	1872	16
Central-line associated blood stream infection	0 (n/a)	457	1
Unknown/other anaemia	10 (n/a)	988	1
Unknown/other thrombocytopenia	8 (n/a)	988	1
Lymphopenia	9 (n/a)	988	1
Endocarditis	2 (n/a)	43	1
Wegener's granulomatosis	2 (n/a)	45	1
Myelodysplasia	0 (n/a)	988	1
**Neurological involvement**			
Infectious meningitis	7 (5-9)[f]	1672	17
Brain abscess	2 (n/a)	43	1
Progressive multifocal leukoencephalopathy	0 (n/a)	32	1
Granuloma in brain	0 (n/a)	473	1
Myasthenia gravis	0 (0-1)	1520	3
Multiple sclerosis	0 (0-1)	1022	2
Bell's palsy	1 (0-2)	1000	2
Polyneuropathy	3 (1-6)	1011	2
Epilepsy	6 (2-11)	480	2
Brain cancer	2 (n/a)	47	1
Meningioma	1 (n/a)	75	1
Uveitis	2 (n/a)	988	1
Granuloma in retina	0 (n/a)	455	1
Attention deficit hyperactivity disorder	6 (n/a)	457	1
Anxiety	3 (n/a)	457	1
Developmental delay	7 (n/a)	457	1
Headaches/migraine	17 (n/a)	457	1
Cerebral atrophy	1 (n/a)	248	1
Schizophrenia	1 (n/a)	248	1
**Bone and joint involvement**			
Septic arthritis	4 (1-8)	719	7
Rheumatoid arthritis	3 (2-4)	1922	8
Seronegative arthritis	4 (2-7)	1284	7
Juvenile idiopathic arthritis	1 (0-4)	1160	3
Psoriatic arthritis	0 (0-1)	1022	2
Arthralgia	20 (n/a)	457	1
Osteomyelitis	4 (1-8)	807	6
Multiple myeloma	0 (0-1)	1212	2
Ewing sarcoma	0 (n/a)	224	1
Kaposi's sarcoma	0 (0-1)	707	2
Osteoporosis/osteopenia	10 (1-25)	480	2
Fasciitis	2 (n/a)	43	1
Myeloid sarcoma	1 (n/a)	106	1
Myositis	1 (n/a)	988	1
Mixed connective tissue disease	1 (n/a)	988	1
Scleroderma/CREST	0 (n/a)	988	1
Nasopharyngeal soft tissue sarcoma	8 (n/a)	12	1
Osteochondroma	5 (n/a)	22	1
Polymyalgia rheumatica	3 (n/a)	32	1
Psoas abscess	0 (n/a)	248	1

Sacroiliitis	1 (n/a)	95	1
**Lymph node and spleen involvement**			
Lymphadenopathy	30 (20-42)	2122	13
Non-malignant lymphoproliferation	28 (16-40)	1462	6
Splenomegaly	29 (22-37)	3153	22
Non-hodgkin's lymphoma	4 (3-5)	2698	13
Splenectomy	6 (3-8)	1068	5
Pancreatic cancer	2 (0-7)	478	2
Leukemia	1 (1-2)	1581	4
Granuloma in lymph node	2 (0-5)	928	2
Granuloma in spleen	1 (0-2)	928	2
Granuloma in bone marrow	0 (0-1)	928	2
Waldenstrom's macroglobulinemia	0 (n/a)	248	1
**Endocrinological involvement**			
Thyroid disease (all)	9 (4-16)	2252	14
Autoimmune thyroiditis	4 (2-6)	2215	11
Diabetes mellitus	2 (1-3)	1523	8
Thyroid cancer	1 (0-3)	951	3
Addison-Biermer disease	1 (n/a)	77	11
Pituitary gland adenoma	1 (n/a)	77	1
Adrenal tumour	1 (n/a)	71	1
Growth hormone deficiency	25 (n/a)	12	1
Ovarian cancer	0 (n/a)	473	1
**Muco/cutaneous involvement**			
Psoriasis	3 (2-5)	1679	6
Atopic dermatitis	12 (7-18)	1307	6
Alopecia	3 (2-4)	1425	6
Vitiligo	5 (3-8)	2440	12
Warts	5 (4-7)	825	4
Skin cancer	3 (1-5)	1319	7
Skin infections	13 (10-17)	1033	11
Cutaneous abscesses	5 (2-10)	72	6
(Recurrent) herpes zoster	9 (5-14)	1178	9
(Recurrent) herpes simplex	9 (2-10)	404	7
Oral candidiasis	6 (2-11)	1514	9
Genital candidiasis	6 (3-12)	114	2
Urticaria	5 (3-8)	1299	5
Oral/dental infections	4 (3-6)	489	2
Skin granuloma	1 (0-2)	928	2
Severe varicella	4 (2-7)	284	2
Recurrent parotitis	6 (0-3)	279	2
Clubbing	19 (0-73)	138	2
Aphthous lesions	16 (5-31)	147	3
Lichen planus	2 (n/a)	47	1
Neutrophilic dermatosis	23 (n/a)	31	1
Mastocytosis	0 (n/a)	988	1
Angioedema	4 (n/a)	23	1
Behcet's disease	2 (n/a)	43	1
Granulomatous gingival hyperplasia	2 (n/a)	126	1
Granuloma in retina	1 (n/a)	77	1
Oral cancer	0 (n/a)	473	1
Vaginal cancer	0 (n/a)	473	1
**Other clinical manifestations**			

Growth retardation/weight loss	13 (7-22)	759	8
Unexplained granuloma	12 (9-16)	2348	11
Fatigue	39 (n/a)	457	1
Vasculitis	2 (1-3)	1782	6
Systemic lupus erythematosus	1 (1-2)	2288	8

[a]Pathogens were only included in this table if the pathogen was mentioned in ≥2 studies.

[b]According to Chapel et al.

[c]Streptococcus pneumonia 15% (8-23), Haemophilus influenzae 19% (8-33), Moraxella Catharalis 7% (0-19), Staphylococcus aureus 7% (3-12), Mycobacterial infection 1% (0-2), Pneumocystis Jiroveci 1% (0-2), Pseudomonas 6% (2-10), Aspergillus 3% (1-5), Enterobacteriaceae 6% (2-13), Mycoplasma 2% (0-4).

[d]Not only pulmonary.

[e]Giardia intestinalis 13% (7-21), Salmonella species 6% (2-12), Campylobacter species 4% (1-8), Clostridium difficile 2% (1-3), cytomegalovirus 2% (0-7), Cryptosporidium species 1% (0-2), Helicobacter pylori 9% (3-18), Escherichia coli 8% (4-13), Candida species 10% (4-19), Strongyloidiasis 7% (3-13).

[f]Streptococcus pneumonia 3% (2-6), Haemophilus influenzae 1% (0-3).

Abbreviations: COPD, chronic obstructive pulmonary disease; CREST syndrome, calcinosis, Raynaud's phenomenon, esophageal dysmotility, sclerodactyly, telangiectasia; GLILD, Granulomatous-lymphocytic interstitial lung disease; n/a, not applicable.
