# Supplementary material for: Lessons Learned From the Clinical Presentation of Common Variable Immunodeficiency Disorders: A Systematic Review and Meta-Analysis
Source: Front Immunol. 2021 Mar 23;12:620709. doi: 10.3389/fimmu.2021.620709 (PMC8021796; doi:10.3389/fimmu.2021.620709)
Supplement: Supplementary file 6 [file DataSheet_6.pdf]

Supplementary Table 6. Subgroup analyses of clinical manifestations in children versus adults

	Children		Adults	
**Manifestation**	**Pooled proportion (95% CI)**	**I^2**	**Pooled proportion (95% CI)**	**I^2**
**Clinical phenotype[a]**				
Infections only	76 (57-91)	84	38 (27-49)	70
Autoimmunity	14 (5-27)	90	21 (12-30)	94
Polyclonal lymphocytic infiltration	n/a	n/a	25 (21-29)	0
Enteropathy	10 (3-20)	78	14 (10-18)	67
Lymphoid malignancy	5 (2-10)	55	6 (5-8)	0
Malignancy	5 (3-7)	0	16 (11-21)	85
**Respiratory involvement**				
Otitis	55 (45-64)	68	32 (27-37)	42
Sinusitis	62 (50-74)	79	74 (60-86)	91
Upper respiratory tract infection	69 (45-89)	91	79 (66-90)	80
Pneumonia	61 (50-71)[b]	74	66 (51-79)[c]	93
Bronchitis	59 (11-71)	97	72 (38-97)	98
Lower respiratory tract infection	71 (59-82)	0	n/a	n/a
Bronchiectasis	16 (9-25)	81	36 (27-46)	93
GLILD	6 (4-9)	0	33 (13-57)	97
Lymphoid hyperplasia	2 (0-6)	30	11 (2-24)	94
Asthma	3 (5-9)	93	33 (21-47)	94
Allergic rhinitis	17 (3-39)	83	n/a	n/a
Allergy	49 (33-65)	40	n/a	n/a
Mastoiditis	4 (0-15)	81	n/a	n/a
Lung cancer	n/a	n/a	1 (0-2)	46
Interstitial lung disease	6 (1-14)	51	15 (3-35)	90
Pharyngitis/tonsillitis	17 (8-28)	78	n/a	n/a
Conjunctivitis	16 (8-25)	64	n/a	n/a
**Gastrointestinal involvement**				
Chronic diarrhea	17 (11-24)	50	34 (22-48)	93
Gastrointestinal tract infection	35 (18-55)[d]	89	34 (8-65)[e]	98
Inflammatory bowel disease	3 (1-6)	32	18 (6-33)	96
Celiac disease	3 (1-5)	10	2 (1-4)	32
Atrophic gastritis	n/a	n/a	9 (0-26)	87
Stomach cancer	n/a	n/a	2 (0-6)	82
Colorectal cancer	n/a	n/a	1 (0-4)	75
Malabsorption	5 (3-8)	0	11 (0-32)	97
Gastro-esophageal reflux disease	12 (1-30)	90	n/a	n/a
**Glandular tissue**				
Sjogren's syndrome	n/a	n/a	3 (0-8)	82
Breast cancer	n/a	n/a	2 (1-3)	49
**Hepatic involvement**				
Autoimmune hepatitis	2 (0-6)	42	4 (0-12)	92
Viral hepatitis	n/a	n/a	2 (1-5)	69
Liver cancer	n/a	n/a	1 (0-1)	44
Unexplained hepatomegaly	21 (2-50)	96	n/a	n/a
**Urinary tract involvement**				
Urinary tract infection	11 (8-15)	0	15 (12-19)	0
Pyelonephritis	8 (0-22)	70	n/a	n/a

**Hematologic involvement**				
Idiopathic thrombocytopenia	9 (2-21)	87	6 (2-12)	86
Autoimmune cytopenia	15 (4-30)	76	16 (7-27)	67
Autoimmune hemolytic anemia	6 (4-9)	0	5 (2-8)	68
Autoimmune neutropenia	4 (0-11)	83	2 (0-6)	86
Evans syndrome	9 (0-26)	86	n/a	n/a
Pernicious anemia	1 (0-3)	61	1 (1-3)	29
Bloodstream infection	8 (5-12)	29	12 (9-16)	47
**Neurologic involvement**				
Infectious meningitis	7 (2-13)	69	7 (4-9)	29
Bell's palsy	2 (0-17)	78	n/a	n/a
Multiple sclerosis	n/a	n/a	1 (0-1)	6
**Bone and joint involvement**				
Septic arthritis	8 (1-22)	67	n/a	n/a
Rheumatoid arthritis	n/a	n/a	4 (1-7)	64
Seronegative arthritis	2 (0-6)	55	n/a	n/a
Psoriatic arthritis	n/a	n/a	1 (0-2)	57
Osteomyelitis	2 (0-6)	68	n/a	n/a
Kaposi's sarcoma	n/a	n/a	0 (0-1)	0
**Lymph node and spleen involvement**				
Lymphadenopathy	22 (44-48)	96	n/a	n/a
Non-malignant lymphoproliferation	n/a	n/a	27 (12-46)	89
Splenomegaly	20 (6-39)	94	30 (16-46)	95
Non-hodgkin's lymphoma	1 (0-3)	14	5 (4-6)	40
Hodgkin's lymphoma	5 (2-11)	0	1 (0-2)	0
Splenectomy	n/a	n/a	7 (5-10)	17
Leukemia	n/a	n/a	1 (0-1)	16
**Endocrinological involvement**				
Thyroid disease	8 (0-24)	66	29 (12-50)	96
Autoimmune thyroiditis	3 (1-6)	0	6 (2-12)	83
Diabetes mellitus	2 (0-4)	0	4 (0-14)	82
Thyroid cancer	n/a	n/a	1 (0-2)	74
**Muco/cutaneous involvement**				
Psoriasis	1 (0-6)	72	4 (2-7)	49
Atopic dermatitis	16 (12-20)	0	n/a	n/a
Alopecia	1 (0-3)	0	n/a	n/a
Vitiligo	2 (1-4)	0	2 (1-4)	48
Warts	6 (3-9)	0	5 (3-7)	0
Skin cancer	1 (0-3)	28	5 (2-8)	64
Skin infections	16 (9-25)	64	13 (6-23)	73
Recurrent herpes zoster infections	19 (12-27)	0	7 (2-15)	91
Recurrent herpes simplex infections	n/a	n/a	17 (0-47)	96
Oral candidiasis	12 (8-16)	2	6 (0-20)	96
Urticaria	3 (1-6)	28	n/a	n/a
Clubbing	5 (1-12)	49	n/a	n/a
**Other clinical manifestations**				
Growth retardation	19 (15-23)	0	n/a	n/a
Unexplained granuloma	6 (2-12)	53	15 (7-25)	90
Vasculitis	1 (0-5)	47	2 (2-4)	0
Systemic lupus erythematosus	1 (0-9)	75	2 (1-3)	0

[a]According to Chapel et al.
[b]Streptococcus pneumoniae 10% (0-29).
[c]Streptococcus pneumoniae 10% (0-28), Haemophilus influenzae 8% (4-14), Mycobacterial infection 1% (0-2), Pneumocystis Jiruveci 1% (0-2), Pseudomonas 2% (1-5).
[d] Giardia intestinalis 13% (7-20), Salmonella species 8% (2-18), Campylobacter species 5% (0-14).
[e]Giardia intestinalis 12% (0-32), Salmonella species 3% (0-12), Campylobacter species 3% (0-10), Clostridium difficile 2% (0-3), Cryptosporidium species 1% (0-2), Helicobacter pylori 6% (1-12).
