# Supplementary material for: Lessons Learned From the Clinical Presentation of Common Variable Immunodeficiency Disorders: A Systematic Review and Meta-Analysis
Source: Front Immunol. 2021 Mar 23;12:620709. doi: 10.3389/fimmu.2021.620709 (PMC8021796; doi:10.3389/fimmu.2021.620709)
Supplement: Supplementary file 7 [file DataSheet_7.pdf]

eSearch: Electronic database search strategy

A. **Embase search strategy for common variable immunodeficiency disease (last conducted January 2019)**
   1. (common variable immunodeficiency OR CVID OR late onset hypogammaglobulinemia OR late onset hypogammaglobulinaemia).ti,ab,kw
   2. AND English/lim
   3. AND 1999-2019/py
   4. NOT (animal cell/de OR animal experiment OR case report OR human cell OR human tissue OR in vitro study OR model OR nonhuman OR a case of:ti)
   5. NOT (human immunodeficiency virus infection OR acquired immune deficiency syndrome OR hiv)
   6. NOT (in vitro OR human cell OR human tissue OR mutation OR cellular).ti,ab,kw

B. **Cochrane search strategy for common variable immunodeficiency disease (last conducted January 2019)**
   1. CVID OR common variable immunodeficiency
   2. NOT (HIV OR AIDS OR human immunodeficiency virus OR acquired immune deficiency syndrome)

C. **PubMed search strategy for common variable immunodeficiency disease (last conducted January 2019)**
   1. sign [Journal] OR sign [All Fields] OR diagnosis [Subheading] OR diagnosis [All Fields] OR diagnosis [MeSH terms] OR signs [All Fields] OR symptoms [All Fields] OR symptom (All Fields] OR clinical [All Fields] OR Feature [All Fields] OR present [All Fields] OR characteristic [All Fields] OR manifestation [All Fields]
   2. AND (common variable immunodeficiency OR CVID OR common variable immunodeficiency disorders OR late onset hypogammaglobulinaemia OR late onset hypogammaglobulinemia).ti,ab
   3. AND 1999/01/01 [PDAT]
   4. AND humans [MeSH Terms]
   5. AND English [lang]
   6. NOT (hiv [MeSH Terms] OR hiv [All Fields] OR human immunodeficiency virus [All Fields] OR acquired immunodeficiency syndrome [All Fields] OR acquired immunodeficiency syndrome [MeSH Terms] OR (acquired [All Fields] AND immunodeficiency [All Fields] AND syndrome [All Fields]) OR aids [All Fields]
   7. NOT (case reports [Publication type] OR case report [All Fields])
